# A theory of resistance to multiplexed gene drive demonstrates the significant role of weakly deleterious natural genetic variation

**DOI:** 10.1073/pnas.2200567119

**Published:** 2022-08-01

**Authors:** Bhavin S. Khatri, Austin Burt

**Affiliations:** ^a^Department of Life Sciences, Imperial College London, Ascot SL5 7PY, United Kingdom;; ^b^Chromosome Segregation Laboratory, and Mechanobiology and Biophysics Laboratory, The Francis Crick Institute, London, NW1 1AT, United Kingdom

**Keywords:** gene drive, resistance, multisite resistance, standing variation, population rescue

## Abstract

Evolution of resistance is a major barrier to successful deployment of gene-drive systems to suppress natural populations, which could greatly reduce the burden of many vector-borne diseases. Multiplexed guide RNAs (gRNAs) that require resistance mutations in all target cut sites are a promising antiresistance strategy since, in principle, resistance would only arise in unrealistically large populations. Using stochastic simulations that accurately model evolution at very large population sizes, we explore the probability of resistance due to three important mechanisms: 1) nonhomologous end-joining mutations, 2) single-nucleotide mutants arising de novo, or 3) single-nucleotide polymorphisms preexisting as standing variation. Our results explore the relative importance of these mechanisms and highlight a complexity of the mutation–selection–drift balance between haplotypes with complete resistance and those with an incomplete number of resistant alleles. We find that this leads to a phenomenon where weakly deleterious naturally occurring variants greatly amplify the probability of multisite resistance compared to de novo mutation. This key result provides design criterion for antiresistance multiplexed systems, which, in general, will need a larger number of gRNAs compared to de novo expectations. This theory may have wider application to the evolution of resistance or evolutionary rescue when multiple changes are required before selection can act.

## Significance

CRISPR-based gene drives have the potential for controlling natural populations of disease vectors, such as malaria-carrying mosquitoes in sub-Saharan Africa. If successful, they hold promise of significantly reducing the burden of disease and death from malaria and many other vector-borne diseases. A significant challenge to success is the evolution of resistance. Here, we develop a theory of resistance for multiplexed drive, which shows the importance of weakly deleterious naturally occurring genetic variation, whose effect is significantly amplified compared to de novo mutation. These results provide a fundamental basis to estimate how many guide RNAs are required to prevent resistance in the face of natural genetic variation.

Suppression gene-drive systems have the potential to be highly effective for population control of many vectors of human disease, including malaria-carrying mosquitoes, as well as for invasive species control (1). With the recent discovery of the CRISPR–Cas9 gene-editing system (2), such drives can be realized by targeting specific sequences for insertion of a drive construct into a gene of importance; during meiosis, this converts a very large fraction of gametes carrying wild-type alleles to this drive construct, thereby increasing transmission of the drive over and above random Mendelian segregation. If the drive construct only has significant fitness effects as a homozygote, it can rise to high frequencies; in particular, if the target gene is necessary for female fertility, this provides an effective way to suppress the population (3, 4). An important question is how resilient gene-drive systems are to the evolution of resistance (1, 5–7), which could arise via single-nucleotide mutations or single-nucleotide polymorphisms (SNPs) or by an imperfect end-joining repair process, called nonhomologous end joining (NHEJ) (3, 8). Although in caged populations, resistance to suppression has been observed via indels induced at a nonconserved target site within a gene critical for female fertility (3), drive systems where the chosen target site has high conservation have been found to be resilient (4). However, it is an open question as to how resilient gene-drive systems are to the evolution of resistance in natural populations, which can have very large population sizes (9).

Although empirically, we understand well the rate at which these processes occur in individuals, from characterization of the mutation rate (μ) and the net NHEJ rate (ν), both per generation, it is also crucially important to know 1) the population size and 2) the fraction of potential resistance mutants that are actually functional. Rather than the per-individual rate, the population-level mutation rate is critical, as it measures how likely such mutations are to arise in the population (5–7). For example, if a resistance mutant is produced per individual at rate $10^{-7}$ and the population size is $N=10^{4}$, the population-level mutation rate is $10^{-3}$, and it will typically take 1,000 generations before a single resistance mutant is generated in a population, and so resistance is unlikely to arise, given that drive typically acts to suppress a population on a timescale of much less than 100 generations; on the other hand, if $N=10^{8}$, 10 such mutants are generated every generation, and so resistance mutants with a selective advantage compared to drive are very likely to arise and fix before population elimination.

A promising antiresistance strategy is to exploit multiple redundant target sites for cleavage by the nuclease (1, 7, 10, 11). If there are multiple guide RNAs (gRNAs) or target cut sites within a single locus, since a single successful cut, followed by homology directed repair, is sufficient for the drive to be copied, all sites must develop resistance mutants in order for the non-Mendelian transmission of drive to fail. This aims to reduce the individual rate of resistance to such small numbers that typically, unrealistic population sizes would be required to achieve a large population-level rate of resistance generation. Although multiplexing has been demonstrated to work in cage trials (12–14), extrapolation of these results to large natural populations is uncertain. Using a deterministic model, Noble et al. (11) showed that multiplexed strategies can allow drive to overcome resistance on short timescales; however, as population dynamics were not considered, in such infinite population-size models, resistance will always arise eventually for any fertile allele. Beaghton et al. (5) explored explicit population dynamics and finite population stochasticity and the probability of resistance using branching process theory in the context of the Y-drive suppression strategy, and Marshall et al. (7) used more complex and realistic stochastic simulations to examine how resistance interplays with population size and the rate of generation of NHEJ mutants. Both of these works showed that large natural population sizes reduce the maximum permissible rate of resistance mutants. Using heuristic arguments, Marshall et al. then argued that relatively modest degrees of multiplexing *m* could prevent resistance from arising. These considerations assume that for multiplexed drive systems, cleavage is independent at each site, while in practice, cleavage may be simultaneous (15, 16) and/or dependent on dosage of Cas9 (16); simultaneous cleavage should be less relevant for suppression drives, as these would generate large deleterious deletions. In addition, none of these works have explored the role of standing variation, though more recently, Lanzaro et al. (17) showed that higher levels of standing variation of resistance variants increase the probability of resistance to various gene-drive strategies, including suppression drives, though crucially, they did not look at the role of population size and multiplexing.

On a theoretical level, there are a number of works in the population-genetic literature relevant to resistance arising in suppression drives (18–21) that predict that population rescue or adaptation in response to an environmental change, in general, is more likely from standing variation compared to de novo mutations arising after the change. Although simple theoretical arguments for the role of standing variation with multisite evolution have been considered using branching-process theory (22), they assumed that the initial frequency of mutants are fixed at their deterministic mutation–balance frequencies. However, stochastic theory (21) shows that this overestimates the role of standing variation, since by averaging over the allele frequency distribution before the change, the advantage over de novo mutation is only weakly logarithmic in the fitness cost and, in general, not very significant. There is currently no theory that addresses the question of the role that standing variation plays, with its full allele-frequency spectrum, in determining the probability of resistance for a multisite evolutionary system like multiplexed gene drive, and this is an important question we address here.

In this work, we develop fully stochastic Wright–Fisher simulations of multiplexed drive to analyze the role of population size in more detail for different degrees of multiplex *m* and for more general scenarios of resistance not previously considered, including, as we shall see, the critical role of standing variation in multiplexed drives. We exclusively explore the population-rescue scenario, where drive is introduced at sufficiently large frequency that the population will be eliminated without resistance. Multiplexing means that the critical population sizes required for resistance can be very large, yet the resistance mutants still arise at very small frequency, requiring a stochastic description of their establishment; to this end, we develop a very accurate Gaussian–Poisson approximation to the multinomial distribution to allow Wright–Fisher sampling in almost arbitrarily large population sizes. We use these simulations to assess the modes of resistance arising for both NHEJ mutants and SNP mutants, where the latter can arise by de novo base-pair mutation or preexist in the population before the introduction of drive as standing genetic variation. Importantly, we also allow each type to be functional or nonfunctional. Our key finding shows a more complex structure of standing variation with multiple gRNAs; this results in a power-law dependence of the critical population size for resistance to arise on the fitness cost of standing variation, compared to the weak logarithmic fitness effects for a single gRNA. This leads to an extreme sensitivity of resistance on the fitness cost, greatly amplifying the probability of resistance, compared to de novo, for weakly selected alleles as the number of target sites and gRNAs increases. As well as being important to predict the success of multiplexed drive systems in preventing resistance at a given population size, we suggest that this result has wide applicability for understanding resistance due to standing variation for a range of multisite evolutionary systems, including the question of vaccine escape.

## Results

Our main results are the probability of resistance arising as a function of the effective population size *N*, for one, two, or three gRNAs. We define four allele types at each target site: wild type, W; functional resistant, R; nonfunctional resistant, N; and drive, D. For *m* > 1, the haplotype of alleles at each of the target sites on each chromosome are some mosaic of these basic alleles (e.g., WRN), except that there cannot be a mosaic with D since drive is either successfully copied or not. A fully resistant haplotype is one that has either R or N at all *m* sites. We will explore the probability of resistance as a function of three main parameters: the fraction of NHEJ mutants that are functional (β), the fraction of de novo mutations that are functional (ξ), and the heterozygous fitness cost (in the absence of drive) of functional resistance (σ) (assumed to be the same whether it was derived from de novo mutation or NHEJ); the remaining fraction 1−β of NHEJ mutants, or 1−ξ of de novo SNPs, are assumed nonfunctional. For simplicity, we assume that all resistant mutations, whether functional or not, completely abolish cleavage, so resistance is complete, and there are no effects of recombination between target sites, or off-target effects (23, 24). The parameters used in the model are summarized in Table 1 with typical values used in the simulations.

**Table 1.** Table of model parameter values and typical values

Typical value	Parameter	Description
ϵ	Cleavage efficiency	0.95
ν	Rate of NHEJ events	0.05
μ	Mutation rate	$5.4\times 10^{-8}$
*s*	Fitness cost of nonfunctional	1
	homozygotes (D or N)	
*h*	Dominance coefficient of W/D	0.3
*h_N_*	Dominance coefficient of	0.02
	nonfunctional resistance (N)	
*N*	Effective population size	Varied
σ	Fitness cost of functional	Varied
	resistance (R)	
β	Fraction of functional NHEJ mutants	Varied
ξ	Fraction of functional	Varied
	single-nucleotide mutations	
*m*	Number of gRNAs/target sites	Varied

All efficiencies/rates are per generation per individual per target site in an independent sites model (*Materials and Methods* and *SI Appendix*). The target site mutation rate is calculated assuming a site of length 18 bp and nucleotide mutation rate of $3\times 10^{-9}$ taken for *Drosophila* (25).

Overall, we find that the probability of resistance has a universal sigmoidal behavior, which is of the form

[1] $$p=1-e^{-N/N^{*}},$$

where for small population sizes, $N\ll N^{*}$ resistance is very unlikely, while for sufficiently large population sizes ($N\gg N^{*}$), resistance arises with near-100% certainty. We use the simulations to develop a heuristic theory to characterize how $N^{*}$ depends on the parameters β,ξ,σ,N and then focus, in particular, on contours in the parameter space of these variables, where the probability of resistance is *p* = 0.05, or 5%.

The results are organized by four different conditions: 1) NHEJ mutations only; 2) de novo SNP mutants only; 3) standing variation of SNP mutants in mutation-selection balance; and 4) where all of the first three mechanisms are possible, where for each, we vary the fraction of functional NHEJ mutants β, the fraction of de novo SNPs ξ, and the fitness costs of functional mutants (NHEJ or SNP) as appropriate.

All the results are determined from running 500 replicate simulations. For each set of parameters, we determine whether resistance to the drive construct arose using a resistance criterion that the sum of the frequency of resistance alleles/haplotypes reaches 0.95: for example, when the frequency of R and N, for a single gRNA, is greater than 95%, or for two gRNAs, when the frequency of RR, RN and NN is greater than 95%. We also examine the time to resistance, given that resistance arises, in *SI Appendix*. In all simulations, drive is introduced at a frequency of 0.1 in males.

### Probability of Resistance: NHEJ Only and Single-Nucleotide De Novo Mutants Only.

**Fig. 1.**
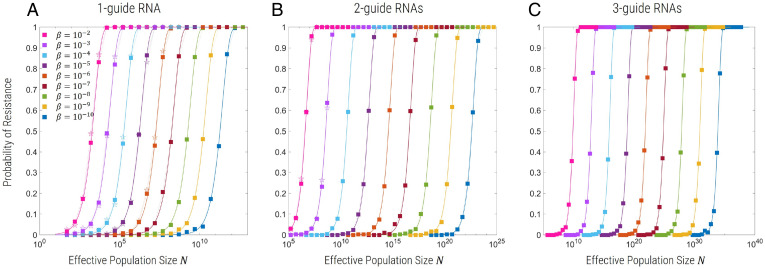
Probability of resistance evolving as a function of effective population size (*N*) for m=1 (*A*), 2 (*B*), or 3 (*C*) gRNAs, where resistance can only arise due to functional NHEJ mutants. R alleles have a fitness cost of σ=0.01 relative to the wild type. Curves of different color correspond to different values of β, where squares correspond to simulations using the hybrid Poisson–Gaussian approximation and open pentagrams to simulations using exact multinomial sampling.

For both NHEJ and de novo single-nucleotide mutants, we find that the simulations of the probability of resistance as a function of population size can be fit very accurately with a sigmoidal form $p=1-e^{-N/N^{*}}$. For NHEJ only, we plot in Fig. 1 p vs. *N* for *m* = 1 gRNA (Fig. 1*A*), *m* = 2 gRNAs (Fig. 1*B*), and *m* = 3 gRNAs (Fig. 1*C*) for the standard parameters outlined above, but where the cost of functional resistance mutants is σ=0.01. Each set of simulation data with square symbols of a given color corresponds to a different value of β, the fraction of functional NHEJ mutants; as β decreases, $N^{*}$ increases, as we would expect, and the probability of resistance decreases for a given *N*. These results (Fig. 1) (square symbols) are obtained by using the hybrid Poisson–Gaussian approximation to the multinomial distribution (*Materials and Methods*), which allows Wright–Fisher simulations at very large population sizes; to check that this approximation works well, the results with pentagram symbols represent simulations at smaller population sizes using multinomial generated random numbers, for which we see very good agreement with the Poisson–Gaussian approximation. We find equivalent sigmoidal curves for de novo SNPs only, where $N^{*}$ increases for decreasing ξ. Again, for NHEJ only, in *SI Appendix*, we show typical time series of the allele frequencies and population dynamics, showing population extinction for $N<N^{*}$ and population recovery and resistance for $N>N^{*}$, when the resistance allele/haplotype R, RR, or RRR fix in the population, for *m* = 1, *m* = 2, and *m* = 3, respectively (*SI Appendix*, Fig. S3).

The dependence of $N^{*}$ on β and ξ for NHEJ and de novo SNPs, respectively, are similar, but with a crucial difference that highlights a difference in the dynamics of multiplex allele generation. For NHEJ, we find:

[2] $$N_{n}^{*}=\frac{1}{4\gamma_{n}{\left(\epsilon \nu \beta \right)}^{m}};$$

and that $\gamma_{n}\approx 0.2$ for all values of *m* (see *SI Appendix* for details), and we see that the fit is excellent, as shown in Fig. 1. This form of $N^{*}$ indicates that once the population-level rate of producing functional resistance mutants $∼N{\left(\epsilon \nu \beta \right)}^{m}$, which requires *m* copies of R, is sufficiently large, then the probability of resistance is large; more specifically, at the critical population size $N=N_{n}^{*}$, when the probability of resistance is large, the population-level rate of generating functional NHEJ mutants is $2N{\left(\epsilon \nu \beta \right)}^{m}\approx \frac{1}{2\gamma_{n}}=2.5∼1$ per generation. As we show in *SI Appendix*, this particular form with a single fixed $\gamma_{n}$ independent of *m* arises when the rate of generation of *m*-fold resistance mutants concurrently is faster than generating them sequentially, which we show is generally the case when cleavage is efficient (1−ϵ≪1). To be clear, both pathways, concurrent and sequential generation of *m*-fold mutants, have a rate that scales as ${\left(\epsilon \nu \beta \right)}^{m}$, but they have different prefactors determining their relative importance.

On the other hand, for de novo SNPs $N^{*}$ takes the following similar functional form:

[3] $$N_{d}^{*}=\frac{1}{4\gamma_{m}{\left(\xi \mu \right)}^{m}},$$

but unlike for NHEJ mutants, the fitting constant $\gamma_{m}$ is not independent of *m*, and we show in *SI Appendix* that it is of the form $\gamma_{m}=s_{b}\tau^{m}$, which arises when multifold resistance alleles arise sequentially, rather than concurrently; here, *s_b_* is an effective beneficial selection coefficient for the resistant haplotype with *m* functional resistant alleles, and τ is the timescale over which these resistant mutants accumulate. By fitting the simulation data, we find that $\gamma_{m}=\{0.76,6,55\}$ for m={1,2,3}, respectively, which corresponds to $s_{b}\approx 0.09$ and τ≈8.5 generations, which, pleasingly, is a time consistent with the timescale over which drive increases to high frequency. As the rate per individual of generating functional resistance at all *m* sites is ${\left(\xi \mu \right)}^{m}$, this means that the population-level rate needed for the probability of resistance to be large is $2N{\left(\xi \mu \right)}^{m}\approx \frac{1}{2\gamma_{m}}\approx \{0.658,0.083,0.009\}$ per generation, for m={1,2,3}, respectively.

**Fig. 2.**
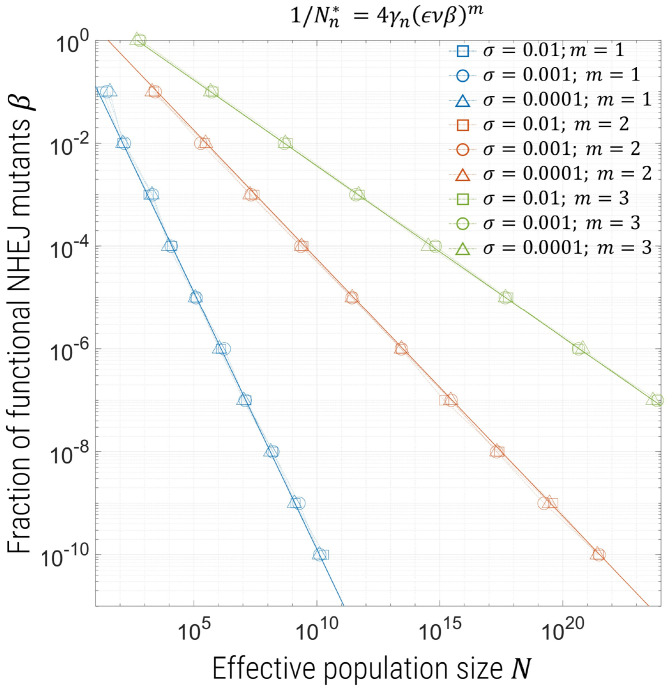
Contours for probability of resistance *p* = 0.05 as a function of *N* and β for *m* = 1 (blue), 2 (red), or 3 (yellow) gRNAs. Different symbols correspond to interpolated values from simulations for different fitness costs for functional mutants: square symbols, σ=0.01; triangle symbols, σ=0.001; circle symbols, σ=0.0001; and the solid lines are plots of $p=1-e^{-N/N_{n}^{*}}$ for $\gamma_{n}=0.2$.

**Fig. 3.**
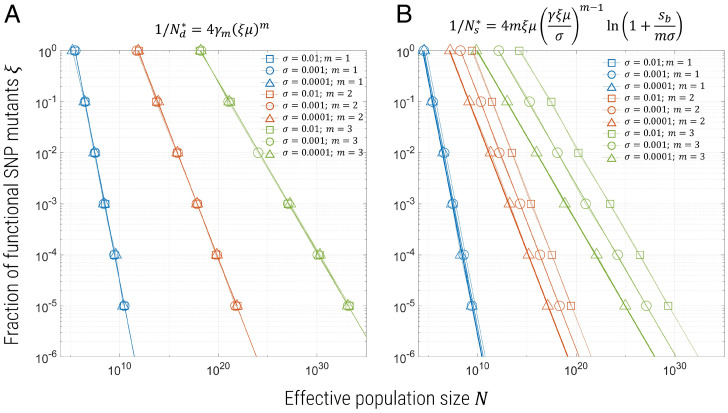
Contours for probability of resistance *p* = 0.05 as a function of *N* and ξ for *m* = 1 (blue), 2 (red), or 3 (yellow) gRNAs. *A* corresponds to simulations with de novo generation of SNP mutants only, while *B* shows simulations with both de novo and preexisting SNP mutants. Different symbols correspond to simulations for different fitness costs for functional mutants: square symbols, σ=0.01; triangle symbols, σ=0.001; circle symbols, σ=0.0001; and the solid lines plot $p=1-e^{-N/N^{*}}$, with $N^{*}=\{N_{d}^{*},N_{s}^{*}\}$ for *A* and *B* given by Eqs.** 3** and **4**, respectively, where for *A*, $\gamma_{m}=\{0.76,6,55\}$, and for *B*, γ=2 and $s_{b}=0.5$.

For both NHEJ and de novo SNPs, we can summarize this information in Figs. 2 and 3*A* by plotting contours of *p* = 0.05 on axes of β vs. *N*, or ξ vs. *N*, where the region to the left of the contour indicates values of these parameters for which we expect the probability of resistance to be ≤0.05. As indicated by Eqs. **2** and **3**, we see that these contours are a power law $∼\beta^{-m}$ and $∼\xi^{-m}$, respectively. The solid lines are plots of $p=1-e^{-N/N_{n}^{*}}$ for *p* = 0.05 and $\gamma_{n}=0.2$ in Fig. 2 and $\gamma_{m}=\{0.76,6,55\}$ for m={1,2,3} in Fig. 3*A*. In both figures, the different symbols correspond to different fitness costs of mutants in the presence of wild type, and we see that, here, this has no effect on contours, which is intuitive, as these mutants only become advantageous once drive has nearly fixed, and their effective frequency-dependent selection coefficient has only a weak dependence on σ, the cost in the presence of wild type only.

To summarize, the key results for NHEJ vs. de novo SNPs are that:

• The critical population size for resistance for both NHEJ and de novo SNPs scales as the inverse of the rate of generation of resistance mutants in individuals (Eqs. **2** and **3**).
• The mechanism of resistance from NHEJ is dominated by resistance alleles arising at all target sites concurrently, while for de novo SNPs, the resistance alleles arise sequentially.

### Probability of Resistance: Preexisting and De Novo SNP Mutants Only.

We now allow the possibility that functional resistant (R) mutants may exist in a mutation-selection balance before the introduction of drive, by running each replicate simulation for a period of time 1/σ before the introduction of drive, which gives sufficient time for the frequency distribution of the SNP mutants at the time of introduction of drive to have equilibrated (*Materials and Methods*). As in the previous sections, we can very accurately fit curves of the probability of resistance vs. *N*, using the same functional form $p_{s}=1-e^{-N/N_{s}^{*}}$, where now

[4] $$N_{s}^{*}=\frac{1}{4\xi \mu m{\left(\frac{\gamma \xi \mu }{\sigma }\right)}^{m-1}\ln(1+\frac{s_{b}}{m\sigma })};$$

where the fitness cost is σ, before drive is introduced. For *m* = 1, this expression corresponds to the theory of Hermisson and Pennings (21), assuming a fixed beneficial selection coefficient of *s_b_* for the resistance mutant in the presence of the drive allele, which is derived by calculating the average probability of fixation over the distribution of allele frequencies in mutation-selection balance. For sufficiently strong selection 4Nσ>1, this allele frequency distribution is closely approximated by a gamma distribution with shape parameter θ=4Nξμ and rate parameter α=4Nσ. In the case of *m* > 1 gRNAs, the functional form for $N^{*}$ can be heuristically motivated by assuming the frequency distribution is of the same form (gamma distributed), but with a modified rate parameter $\alpha_{m}=4Nm\sigma$ and shape parameter $\theta_{m}∼4N\xi \mu r_{m-1}^{*}∼4N\xi \mu {\left(\xi \mu /\sigma \right)}^{m-1}$, where $r_{m-1}^{*}$ is the frequency of mutants with *m* – 1 R alleles and a single W in mutation-selection balance before drive is introduced. For example, if there are *m* = 2 gRNAs, then $r_{1}^{*}$ is the frequency of RW, while for *m* = 3 RNAs, *r*_2_ is the frequency of RRW. We show in *SI Appendix* that $r_{m-1}^{*}∼{\left(\xi \mu /\sigma \right)}^{m-1}$. $\theta_{m}$ represents the effective mutational flow from *m* – 1 mutants to *m* mutants, which is balanced by negative selection, where each *m* mutant has fitness cost mσ, before the introduction of drive. By averaging the probability of fixation over this distribution, we recover the scaling law for $N_{s}^{*}$ stated above. We do not attempt to calculate an exact theory, but use a fitting parameter γ to represent the scaling between these heuristic considerations and an exact theory.

We first fit the curves of probability of resistance vs. *N* for *m* = 1, where γ does not appear in $N_{s}^{*}$, which gives an approximate value of $s_{b}\approx 0.5$ for all values of ξ (*SI Appendix*). For *m* > 1, we fix *s_b_* to this value and fit the curves of *p*(*N*) for the single fitting parameter γ and find that γ≈2 for both *m* = 2 and *m* = 3, as shown in *SI Appendix*. In all cases, we find that the curves fit the simulation data very well.

We summarize these results by plotting contours of constant probability of resistance *p* = 0.05 as a function of ξ and *N*, as shown in Fig. 3*B*; regions to the left of each curve represent combinations of ξ and *N* for which the probability of resistance is less than 5%. The major effect of preexisting mutations is to very greatly reduce the effective population size needed before resistance arises for more than *m* > 1 gRNAs compared to de novo SNPs. Importantly, unlike for de novo SNPs or NHEJ mutations, there is a significant effect of changing selection, where the probability of resistance increases for decreasing σ because less-harmful mutations will segregate at a higher frequency before release. Hermisson and Pennings (21) showed this to be the case for a single site (*m* = 1) (6, 21), in which case the effect is only weak and logarithmic in σ. However, here, we see for *m* > 1, $N^{*}∼\sigma^{m-1}$, which represents a significant amplification of the role of more weakly selected standing variation in causing resistance.

In summary, the key results for preexisting SNPs are that:

• For m > 1, the more complex mutation–selection–drift balance for multiple target sites (treated heuristically in SI Appendix) means that the critical population size for resistance from preexisting SNPs (Eq. 4) picks up an additional power-law dependence on the fitness cost of these SNPs before the introduction of drive compared to the weak logarithmic dependence for m = 1.
• The probability of resistance for m > 1 is greatly amplified compared to de novo mutation in the presence of weakly deleterious mutation.

### Probability of Resistance: NHEJ, De Novo, and Preexisting SNPs Combined.

**Fig. 4.**
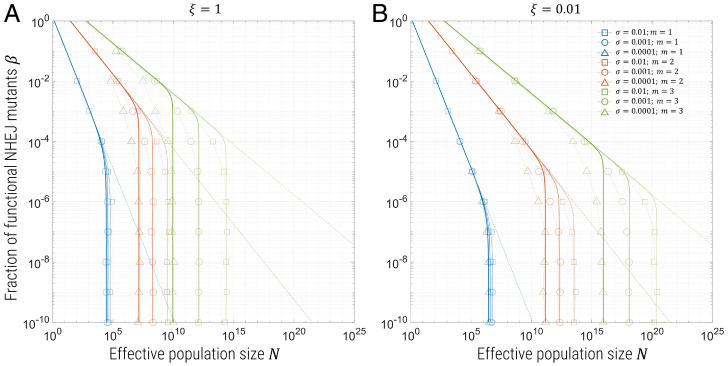
Contours for probability of resistance *p* = 0.05 for β vs. *N* and ξ=1 (*A*) and ξ=0.01 (*B*), for simulations including NHEJ, de novo, and preexisting SNPs. Blue symbols represent *m* = 1, red *m* = 2, and yellow *m* = 3, where the different symbols present different fitness cost σ of mutants in the presence of wild type, as shown in the legend. The solid lines are plots of $1-e^{-N/N^{*}}$, for $1/N^{*}=1/N_{n}^{*}+1/N_{s}^{*}$–which assumes the probability of resistance from NHEJ and SNPs are independent–for exactly the same values of the γ parameters in the previous sections/plots, where the thick lines correspond to σ=0.0001, intermediate thickness to σ=0.001, and thin lines to σ=0.01. The dotted lines are the same as the solid lines from Fig. 2, which are the contours of *p* = 0.05 for NHEJ only.

Finally, we combine all three mechanisms by which resistance can arise to assess their relative importance as a function of varying β, ξ, and σ. The summary of these results is shown in Fig. 4, for contours of *p* = 0.05 for β vs. *N*, for the case of all SNPs being functional (ξ=1; Fig. 4*A*) and 1% functional SNPs (ξ=0.01; Fig. 4*B*). The same broad trends as observed in the simulations of the previous sections are seen, where decreasing β increases the range of population sizes for which resistance is improbable; the asymptotic power-law behavior seen as β→1 is the same as the simulations with NHEJ only, indicated by the dotted lines for each value of *m*, which are the solid lines in Fig. 2. However, in the presence of preexisting SNPs, we find that for sufficiently small β, this effect plateaus and the 5% contours are constant with respect to *N*, as the dominant and more rapid mechanism of resistance becomes SNPs from standing variation. We also find that increasing ξ decreases the population size at which resistance arises at 5%, given by a uniform shift of these curves to the left, as seen by comparing Fig. 4*A* to Fig. 4*B*. The solid lines in each plot correspond to assuming that the probability of resistance from NHEJ mutations and preexisting (and de novo) mutations are independent: $p_{\mathrm{res}}=1-(1-p_{n})(1-p_{s})=1-e^{-N/N^{*}}$, where

[5] $$\begin{array}{l} \frac{1}{N^{*}}=\frac{1}{N_{n}^{*}}+\frac{1}{N_{s}^{*}}\\ =4\gamma_{n}{\left(\epsilon \nu \beta \right)}^{m}+4\xi \mu m{\left(\frac{\gamma \xi \mu }{\sigma }\right)}^{m-1}\ln\left(1+\frac{s_{b}}{m\sigma }\right). \end{array}$$

The plots use the parameters $\gamma_{n}=0.2$ and $\gamma_{s}=2$, as derived from fitting in the previous sections, where the different line thicknesses correspond to different values of σ; we see that, asymptotically, for either β→1 ($1/N_{n}^{*}\gg 1/N_{s}^{*}$) or β→0 ($1/N_{n}^{*}\ll 1/N_{s}^{*}$), where NHEJ mutations are dominant and SNPs are dominant, respectively, the solid lines match the simulation data very well, as expected. However, for the intermediate regime, where NHEJ mutations and SNPs from standing variation are equally dominant, there is a mismatch, which indicates that the assumption that resistance from NHEJ and SNPs are independent is relatively poor. This is as expected, since this heuristic ignores the mosaic nature in which resistance arises at multiple target sites, where, for example, at one site, resistance may have arisen by standing variation and the other two by NHEJ; nonetheless, we see this heuristic gives a good guide to the probability of resistance in the presence of both NHEJ and standing SNP variation.

In summary, when we examine all possible mechanisms of resistance arising for multiplex drive:

• If the rate of producing functional NHEJ mutants per individual is large, such that $N_{n}^{*}\ll N_{s}^{*}$, then resistance is dominated by NHEJ.
• Conversely, if the rate of producing NHEJ mutants is small, such that $N_{n}^{*}\gg N_{s}^{*}$, then resistance is dominated by standing genetic variation

### How Many gRNAs Are Needed to Prevent Resistance?.

An important practical question that arises is how many gRNAs are sufficient to prevent resistance to a high probability. Assuming a regime of β, where NHEJ mutations are dominant, using Eqs. **1** and **2**, we can find the required number of gRNAs to keep the probability of resistance to less than or equal to *p*:

[6] $$m>\frac{\ln\left(\frac{1}{4\gamma_{n}}\ln\left(\frac{1}{1-p}\right)\right)-\ln(N)}{\ln(\epsilon \nu \beta )}.$$

This result is similar to equation 2 of ref. 7, the main difference being that it is derived by approximately solving the dynamics of resistance-allele generation, using their probability of fixation (*SI Appendix*) and explicitly accounting for a fraction β of functional NHEJ mutants.

We can find an equivalent expression for de novo SNPs using Eq. **3**, but since our results indicate that standing variation will always be at least as important as de novo generation of SNPs, we directly consider the constraint on *m* for standing variation. Using Eqs. **1** and **4**, we can, in principle, calculate the minimum number of gRNAs *m* required to prevent resistance to a specified probability. Eq. **4** is transcendental, but if we replace the weak *m* dependence in the logarithm ($\ln(1+\frac{s_{b}}{m\sigma })\to \ln(1+\frac{s_{b}}{\sigma })$), then, for σ≫ξμ, we find the approximate expression:

[7] $$m>\frac{1}{\ln(\frac{\gamma \xi \mu }{\sigma })}W_{-1}\left(\frac{\gamma }{4N\sigma }\frac{\ln(\frac{1}{1-p})\ln(\frac{\gamma \xi \mu }{\sigma })}{\ln(1+\frac{s_{b}}{\sigma })}\right)\text{​},$$

where $W_{k}(x)$ is the Lambert *W* function, which are solutions of the equation $we^{w}=x$.

When both mechanisms of resistance are possible, the effective critical population size $N^{*}$ is a combination of $N_{n}^{*}$ and $N_{s}^{*}$. As shown in Fig. 4, Eq. **5** is a reasonable approximation, but it is difficult to invert this expression to find how many gRNAs are needed to prevent resistance to a certain probability *p*. Here, we instead estimate this critical population size for different values of *m*, β, ξ, and σ by reading off Fig. 4 or by extrapolation using the approximation in Eq. **5**, which we can compare to whichever target population size we have in mind for an application of drive. We can examine two different extremes: 1) $\beta =10^{-4}$, which corresponds to assuming that functional NHEJ mutants are quite rare, as we might expect given the expectation that NHEJ will tend to produce significant genetic changes like multiple base-pair insertions and deletions; and 2) $\beta =10^{-2}$, which is more pessimistic, should in fact NHEJ more readily produce less significant genetic changes, which are more likely to be functional. For the fraction of functional SNPs, we assume a worst case that ξ=1 and the scenario that ξ=0.01, which roughly corresponds to, on average, a single functional mutant in all the 3*L* one-step mutants about the wild-type sequence in a target site of size *L* = 18 bp (values of ξ≪0.01 effectively correspond to ξ=0). To calculate *N* corresponding to when resistance is equal to 5%, we can read off from Fig. 4 or use our extrapolation of the simulations using Eq. **5** to different values of σ. These population sizes are shown in Table 2.

**Table 2.** Effective population sizes *N* at which a probability of resistance *p* = 0.05 is obtained from simulations and theory for different numbers of gRNAs *m* and different selection coefficients σ, for the fixed value of fraction of functional mutants ξ=1 and ξ=0.01

** **	*m* = 1	*m* = 2	*m* = 3	*m* = 4 *
$\mathrm{\xi =}1,\;\mathrm{\beta =}10^{--4}$				
$\sigma =10^{-6}$ *	$8\times 10^{3}$	$9\times 10^{4}$	$6\times 10^{5}$	$4\times 10^{6}$
$\sigma =10^{-4}$	10^4^	$3\times 10^{6}$	$4\times 10^{9}$	$7\times 10^{12}$
$\sigma =10^{-2}$	10^4^	$4\times 10^{8}$	$2\times 10^{13}$	$2\times 10^{19}$
$\mathrm{\xi =}1,\;\mathrm{\beta =}10^{--2}$				
$\sigma =10^{-6}$ *	100	$7\times 10^{4}$	$6\times 10^{5}$	$4\times 10^{6}$
$\sigma =10^{-4}$	100	$7\times 10^{4}$	$3\times 10^{7}$	10^12^
$\sigma =10^{-2}$	100	$3\times 10^{5}$	$5\times 10^{8}$	10^12^
$\mathrm{\xi =}0.01,\;\mathrm{\beta =}10^{--4}$				
$\sigma =10^{-6}$ *	10^4^	$7\times 10^{8}$	$6\times 10^{11}$	$4\times 10^{14}$
$\sigma =10^{-4}$	10^4^	$4\times 10^{8}$	$3\times 10^{13}$	10^20^
$\sigma =10^{-2}$	10^4^	$3\times 10^{9}$	$3\times 10^{17}$	10^20^
$\mathrm{\xi =}0.01,\;\mathrm{\beta =}10^{--2}$				
$\sigma =10^{-6}$ *	100	$3\times 10^{5}$	$6\times 10^{8}$	10^12^
$\sigma =10^{-4}$	100	$3\times 10^{5}$	$3\times 10^{9}$	$2\times 10^{13}$
$\sigma =10^{-2}$	100	$3\times 10^{5}$	$6\times 10^{8}$	$2\times 10^{13}$

The starred values indicate population sizes extrapolated from simulations results using heuristic theory (Eq. **5**).

### Time to Resistance.

In *SI Appendix*, we also examine the mean time taken for resistance to arise, given that resistance arose, where resistance corresponds to the establishment and then fixation of a functionally resistant allele. Specifically, we define the time to resistance to be when the sum of the frequencies of resistance haplotypes exceeds or equals 0.95; this high threshold is a good indication of the time to fixation, which is generally much larger than the time to establishment of resistant haplotypes.

We find across all simulations, very consistently, that the mean time is between 45 and 65 generations, where, as expected in general for large values of β, we have shorter times, since the rate of producing NHEJ mutants is larger, and, as the population size is increased, the time to resistance increases. Broadly, we find that if variants are more deleterious before the introduction of drive and/or as the population size increases, the time to resistance is longer. These results point to the fact that the time for resistance to arise is conditioned on resistance arising, and so all the variants that are destined to give resistance must be generated, or preexist, before the population is eliminated; population elimination happens on a timescale set by the dynamics of drive replacing the wild type–which does not change for different simulations performed in this paper–and by the size of the initial population, where larger populations take a logarithmically longer time to elimination (on average).

## Discussion

Understanding and overcoming resistance in suppression-drive systems is a major obstacle to successful control of natural populations that are vectors for disease, such as malaria (3, 4). As this work and previous research show, population size is a key determinant of the probability of resistance (5–7). In particular, we have highlighted the role of the critical population size $N^{*}$ as a useful summary measure of the probability of resistance compared to some target natural population size. Multiplexing of gRNAs, such that resistance is required in all gRNAs, is a promising antiresistance strategy, as it aims reduce the individual rate of resistance sufficiently that $N^{*}$ is much greater than the target population size. Our results have highlighted five key parameters determining $N^{*}$ and the probability of resistance evolving for multiplexed drive: β, ξ, σ, *m*, and *N*. The expressions we derive from simulations for $N^{*}$ in terms of these parameters inform on the relative importance of NHEJ vs. de novo single-nucleotide mutations vs. standing variation of SNPs. Our key finding is a significant amplification of the role that weakly deleterious standing genetic variation plays in determining resistance in multisite evolutionary systems, compared to de novo mutation. This means, for example, that designing a multiplexed drive system based on the number of gRNAs required for de novo mutation–i.e., at least a couple, for rates of generation of de novo functional resistance, which are of order $∼10^{-8}$ or less–will not prevent resistance in the presence of weakly deleterious standing variation.

### An Estimate of the Number of gRNAs Required for Different General Scenarios.

By judicious choice of target sites, researchers developing gene-drive constructs for population suppression will have some level of control over all of the key parameters identified above except population size. NHEJ typically produces insertion or deletion mutations (3), so choosing a target site that is unable to tolerate length variation will be one way to reduce β, and this would usually be a top priority in choosing a target site. A target site in a region coding for an unstructured loop of a protein might have a β∼1/3 (if 2/3 of indels produce frameshift mutations), but a target site in a more structurally constrained region may have a much smaller β. Hammond et al. (3) have demonstrated that having a single target site that can tolerate length variation quickly leads to the evolution of resistance, even in small populations (N≈600). Though data are scarce, the next most frequent type of mutation produced by NHEJ is presumably single-nucleotide changes at the cut site, and ensuring that those are nonfunctional would be a second priority. Assuming a target population of $N=10^{6}$ and the baseline parameter values, Eq. **6** indicates that under the worst-case scenario of β=1, *m* = 6 gRNAs will be needed to have the probability of resistance arising due solely to NHEJ be less than 0.05, whereas if β can be reduced to 0.01, then only *m* = 3 will be needed, and if β can be reduced further to $10^{-4}$, then only 2 will be needed. If the target population is $N=10^{9}$, then the corresponding values are m={8,4,2} gRNAs for $\beta =\{1,0.01,10^{-4}\}$, respectively.

Single-nucleotide mutations can also arise spontaneously and, if the population is large, may be already present before release. These may also provide resistance against cleavage, particularly if they are near the cut site (26). The likelihood that resistance evolves from these mutations depends on ξ, the fraction of them that are functional (since it is only these that can spread through a population), and on σ, the extent to which those functional resistant mutations reduce fitness (since that affects their frequency in the prerelease population). Again, all else being equal, more functionally constrained sequences will be preferred, having lower ξ as well as lower β, though, if base changes are less likely to be harmful than indels, then ξ will be greater than β. In principle, genetic surveys of sequence variation at the target site may provide useful information on ξ and σ and the probability of resistance evolving through standing variation. Assuming a target population of $N=10^{6}$, baseline parameter values, and $\sigma =10^{-6}$, then using Eq. **7** under the worst-case scenario of ξ=1, *m* = 4 gRNAs are needed to ensure the probability of resistance evolving from de novo mutations and preexisting variation is less than 0.05, whereas just *m* = 2 gRNAs are needed if $\sigma =10^{-4}$ or σ=0.01, which demonstrates the sensitivity to weakly deleterious standing variation. However, if ξ=0.01, then only one gRNA is needed, irrespective of the value of σ. If the target population is $N=10^{9}$, the corresponding values are that m={7,3,2} gRNAs are needed, respectively, for ξ=1 and $\sigma =\{10^{-6},10^{-4},0.01\}$, whereas for ξ=0.01, *m* = 2 gRNAs are needed, regardless of σ.

### Critical Population Size for Resistance for *Anopheles gambiae.*

An important potential application of suppression drives is to control populations of mosquitoes to reduce the burden of malaria on human populations. Recently, the contemporary effective population size for *A. gambiae* in sub-Saharan Africa was estimated as $N∼10^{9}$, using a new method based on analyzing soft sweeps (9). The above considerations of NHEJ and standing variation separately give an indication of how many gRNAs are required to achieve a probability of resistance less than 0.05, in each scenario, but including both mechanisms, we refer to Table 2. Given a target population size of $N=10^{9}$, and assuming ξ=1, these numbers indicate that if resistance alleles are strongly deleterious (σ=0.01) to moderately deleterious ($\sigma =10^{-4}$), then resistance can be prevented with *m* = 3 gRNAs if $\beta =10^{-4}$ or *m* = 4 gRNAs if $\beta =10^{-2}$. However, if the resistance alleles are very weakly deleterious ($\sigma =10^{-6}$), then even four gRNAs are not sufficient, for both values of β, which exemplifies the strong amplification of the probability of resistance in the presence of weakly selected standing variation. On the other hand, if ξ=0.01, which roughly corresponds to, on average, a single functional mutant in all the 3*L* one-step mutants about the wild-type sequence in a target site of size *L* = 18 bp, we can prevent resistance for all values of σ and β with *m* = 3 gRNAs, except for very weakly deleterious mutants ($\sigma =10^{-6}$) and $\beta =10^{-2}$, which requires at least *m* = 4 gRNAs. These considerations highlight the need to empirically determine both β and ξ for putative target sites for multiplex suppression-drive applications, using genetic screens that determine the functionality of NHEJ mutants and by examining SNP variants and their fitness effects from population genomic data.

### Simplifications of Model.

These simulations make a number of simplifying assumptions that are necessitated by modeling the already relatively complex situation of multiplex drive. We assume that all of the nucleotides ξ corresponding to the fraction of functional mutations in each target site, once mutated, completely abolish cleavage and give complete resistance. We also assume that there is no recombination between sites and, related to this, that resistance only arises due to target-site effects; recombination and off-target resistance have been explored by using deterministic modeling (23, 24). We also have not explored the role of varying a number of parameters like cleavage efficiency ϵ, the intrinsic growth rate *R_m_*, or the fitness parameters of drive; some of these have been previously explored in the context of single gRNAs (6, 7), and these works and ours in general indicate that these will have a secondary role compared to the population-scaled rate of NHEJ and de novo mutation. For example, increasing ϵ will tend to increase the rate that drive replaces wild type, and we may expect that one consequence is a quantitative reduction in the probability of resistance, as there is less time to generate mutants and establish before population elimination, but the qualitative results we have presented will not change. However, there is evidence that the overall efficiency of cleavage may diminish when there are large numbers of gRNAs (16). There is also the possibility that homing events may be more error-prone than normal DNA replication and could lead to the loss of function of one or more gRNAs, which we leave to future modeling efforts, but could increase the probability of resistance evolving depending on the rate of such events occurring. We also ignore the role of demographic fluctuations, which would tend to mainly affect neutral variation within a target site, where σ<1/N, and could be much smaller than expected due to historical population bottlenecks (9, 27); for highly conserved sites, this is less likely to play a major role.

On one hand, it is not clear that spatial structure will strongly affect these results, since the population size we consider here should be very close to the census size; whether spatially separated or in a well-mixed system, the number of mutations arising per generation will depend on the total number of individuals in the population. However, what is likely to be different is how quickly a resistance mutation establishes and spreads; in spatially structured populations, selection is effectively weaker (28), reduced by factor $1-F_{ST}$, where $F_{ST}=1/(1+4Nm)$ is Wright’s fixation index for the island model, which means that fixation would be less rapid in a geographically dispersed population. This could lead to an increase in the critical population size $N^{*}$, since at a given population size, the probability of resistance is smaller, as elimination is more likely before the resistance allele becomes sufficiently prevalent in the population. However, this is likely to only have a significant effect in very highly structured populations (very limited migration), since typically, selection for resistance mutants is very large once drive has risen to large frequency. Importantly, in models with spatial structure, even arbitrarily strong gene drives may not eliminate a target population (29–31). This effect can arise if the gene drive causes reductions in population density, which leads to increased inbreeding, which, in turn, reduces the efficacy of the drive (32, 33). If the population is not eliminated, then eventually, one would expect resistance to evolve, though if the population is substantially suppressed, this may take a long time.

### Broader Applications to Evolution of Multisite Resistance.

These results also have broader implications for evolutionary theory, particularly evolutionary mechanisms by which adaptation occurs in response to an environmental change, such as the introduction of drive, or in other contexts, such as resistance to antibiotics or vaccines. Theory for a single site (*m* = 1) in various evolutionary contexts includes the question of which is more important, de novo vs. standing variation for adaptive evolution (21, 27), population rescue (18), or in the context of the evolution of gene-drive resistance (6). All of these studies show that changing the magnitude of the fitness cost σ before the environmental change has a relatively weak effect on the probability of resistance, as borne out by the logarithmic dependence of $N_{s}^{*}$ on σ for *m* = 1 in Eq. **4**. However, a major finding is that for the multiplex drive case, where resistance alleles must evolve at all *m* target sites in order for resistance to arise, there is, in fact, a marked dependence on the fitness of resistance alleles before the introduction of drive. This arises from a complex mutation–selection–drift balance between fully resistant (*m* R alleles) and incomplete resistant (less than *m* R alleles) haplotypes, resulting in a significant amplification of weakly deleterious alleles in their contribution to resistance in a multiplex scenario and a significant reduction in the critical population size with standing variation compared to de novo mutation, as seen in Fig. 3*B*. We can quantify this amplification by calculating the ratio

[8] $$\frac{N_{d}^{*}}{N_{s}^{*}}=\frac{m\gamma^{m-1}\ln\left(1+\frac{s_{b}}{m\sigma }\right)}{\sigma^{m-1}\gamma_{m}},$$

which has values $\frac{N_{d}^{*}}{N_{s}^{*}}\approx \{5,217,6266\}$ for m={1,2,3} and σ=0.01, and $\frac{N_{d}^{*}}{N_{s}^{*}}\approx \{11,5.2\times 10^{4},1.6\times 10^{8}\}$ for $\sigma =10^{-4}$, which are very large amplification factors for *m* > 1 and with consequent implications for the prediction of the probability of resistance for multiplexed gene drives (Note that these very large differences are somewhat hidden in Fig. 3, as the results are plotted on a log scale and over many orders of magnitude). It is interesting to note that if we instead assumed that the frequency of functional resistance mutants at each target site is the mean frequency ξμ/σ expected from mutation-selection balance, this would give $N_{s}^{*}∼{\left(\sigma /\xi \mu \right)}^{m}$ and $N_{d}^{*}/N_{s}^{*}∼1/\sigma^{m}$, which would overestimate the importance of standing variation; the key difference arises from averaging over the distribution of functional resistance mutants at each site, which reduces the critical population size by a factor 1/σ.

These findings may apply more widely to the evolution of resistance or evolutionary rescue when multiple changes are needed for selection to act. Combination therapy is often used in the context of antibiotics (34, 35), antivirals (36), and anticancer treatments (22, 37), and analogous principles are used by vaccine designers and the natural immune system, where multiple epitopes on a virus or other pathogen are targeted (38, 39). All else being equal, combination therapy can be expected to be more effective in preventing the evolution of resistance when resistance to all components of the therapy is needed before fitness differences appear and selection can act. To the extent that this ideal can be achieved, Eq. **4** may be useful in predicting the likelihood of resistance nonetheless evolving.

### Summary.

Overall, our results provide a foundation to understand how resistance arises in multiplexed suppression-drive systems and the paramount role that standing variation plays in greatly amplifying the role of weakly deleterious variation in giving rise to resistance. The results highlight the need to characterize important unknown parameters, such as the fraction of functional mutants at drive target sites, due both to NHEJ mutations and single-nucleotide mutations, which can significantly affect the probability that resistance arises.

## Materials and Methods

We use nonspatial Wright–Fisher stochastic simulations of drive with separate sexes throughout, but with coupling to population dynamics using density-dependent Beverton–Holt growth. The details of these simulations are given in *SI Appendix*. The simulations are stochastic, even when population sizes are very large, because resistant mutations always arise at small frequency, so genetic drift needs to be explicitly considered. Resistance corresponds to the establishment and then fixation of functionally resistant alleles, which we define to be a frequency greater than 0.95. The simulations entail stochastic dynamics with one, two, or three gRNAs, where at each target site, there are four alleles that can occur: W (wild type), D (drive), R (functional resistance), or N (nonfunctional resistance); this means that across an *m*-fold target site, we have an *m*-locus, four-allele population genetic system with no recombination. However, as we assume the drive construct is copied over as a whole, in practice, it is an *m*-locus three-allele system + 1 for the D allele. On a single chromosome, the possible haplotypes (assuming no positional effects) for a twofold system are WW,WR,RR,WN,RN,NN,DD, which is a total of n(n+1)/2+1=(3×4)/2+1=7 haplotypes, and an analogous calculation in *SI Appendix* for *m* = 3 gives a total of 11 haplotypes.

The alleles are assumed to have no effect on male fitness, while fitness effects in females are as follows. W alleles have zero fitness cost, and D and N alleles are deleterious with homozygous fitness cost *s* = 1 and heterozygous fitness costs (when paired with a W allele) of *h* = 0.3 and $h_{N}=0.02$, respectively; the larger dominance coefficient for drive represents potential somatic fitness costs to heterozygotes females, from leaky expression of the Cas-9 protein (4). The heterozygous fitness cost of R, before the introduction of drive, is σ, which we vary in the simulations. When combined in haplotypes, we assume that each target site has independent fitness effects, which means that a single occurrence of an N allele is deleterious. Fitness costs are manifest as reduced female survival. Further details are given in *SI Appendix*.

In W/D heterozygotes (and their multiplexed equivalents discussed in *SI Appendix*), we assume a cleavage efficiency ϵ=0.95 and an NHEJ rate of ν=0.05 approximately representative of the target site in the gene *doublesex* in *A. gambiae* (4). We assume that NHEJ mutants produce functional resistant alleles R with probability β and nonfunctional resistant alleles N with probability 1−β. As a result, D gametes are generated from conversion of W at a rate (1−ν)ϵ, functional resistance alleles R at rate ϵβν, and nonfunctional alleles N at rate ϵ(1−β)ν, while the fraction that remain wild type is 1−ϵ.

We assume that the probability of cleavage at each available target site, given by efficiency ϵ, occurs independently at different sites, so that the fraction of nondriving gametes produced from a genotype with a nondriving allele/haplotype paired with a driver is $\frac{1}{2}{\left[1-\epsilon (1-\nu )\right]}^{m-r}$, where *m* is the number of gRNAs and r≤m is the number of resistant (R or N) sites in the nondriving allele. Thus, when there are multiple gRNAs, the presence of a resistant site gives some protection to the chromosome from being cut, even if one or more cleavable sites remain.

In addition, functional resistance alleles R are generated de novo at rate ξμ, where μ is the mutation rate for the length of site of interest and ξ is the fraction of SNP mutations that are functional, and nonfunctional resistance alleles at rate (1−ξ)μ. We assume $\mu =18\mu_{0}=5.4\times 10^{-8}$, where 18 is the length of each target site and $\mu_{0}=3\times 10^{-9}$ is the base-pair mutation rate measured for *Drosophila* (25).

### Standing Genetic Variation.

To study the effect of standing variation, we run replicate simulations where we allow a burn-in period of 1/σ generations to allow for the population to come to a mutation-selection balance equilibrium. The initial frequency of the various resistance alleles/haplotypes when drive is introduced at *t* = 0 is then implicitly drawn from the mutation-selection balance equilibrium. Note that in the case of *m* > 1, the mutation-selection balance distribution will be complex, with different frequencies for haplotypes carrying different numbers of resistance mutations R.

### Gaussian–Poisson Hybrid Approximation to Generate Multinomial Random Numbers.

In this paper, we run simulations to very large effective population sizes. While it is typical in such a scenario to ignore the stochastic part of the evolutionary dynamics by using deterministic dynamics, this is only accurate if the allele frequencies are large themselves or, equivalently, the number of copies in the population are large (≫1). We are interested in the dynamics of resistance, which, by definition, means that we need to study situations where the allele arises by de novo mutation as a single copy in a single individual, where it must survive genetic drift or exist at very low frequency as standing variation. When there are multiple alleles, particularly when simulating Wright–Fisher evolutionary dynamics, this is accomplished simply by drawing multinomial random numbers. However, when the effective population size is large, this can become increasingly slow. In addition, the maximum population size is restricted to the largest integer that can be stored in a computer; for the GNU scientific library’s implementation of multinomial random number generators, this is limited to 32-bit, which gives a limit of roughly $4\times 10^{9}$.

An alternative approach is to use the multivariate Gaussian approximation to the multinomial distribution:

[9] $$p(n_{1},n_{2},\ldots ,n_{K}|x_{1},x_{2},\ldots ,x_{K},N)$$

[10] $$=\frac{N!}{n_{1}!n_{2}!\ldots n_{K}!}x_{1}^{n_{1}}x_{2}^{n_{2}}\ldots x_{K}^{n_{K}},$$

[11] $$\approx \frac{\exp\left(-\frac{1}{2}{\left(n-Nx\right)}^{T}\Sigma^{-1}(n-Nx)\right)}{\sqrt{{\left(2\pi \right)}^{K}\text{det}(\Sigma )}},$$

where $n={\left(n_{1},n_{2},\ldots ,n_{K}\right)}^{T}$ and $x={\left(x_{1},x_{2},\ldots ,x_{K}\right)}^{T}$ are the vectors of the numbers drawn of *K* alleles and their expected frequency, respectively, and Σ is the scaled covariance matrix of the multinomial distribution, where $\Sigma_{ij}=Nx_{i}(\delta_{ij}-x_{j})$. However, this approximation is poor when for any of the alleles $Nx_{k}∼1$. In this limit, these rare alleles are well-approximated by a Poisson distribution. The approach taken in this paper is therefore to partition the alleles into a rare category, R, if $Nx_{k}\leq 10$ and nonrare if $Nx_{k}>10$, where the former is drawn from independent Poisson distributions, while the latter is from a multivariate Gaussian distribution conditioned on a smaller total population size $N\prime =N-\sum_{k\in R}n_{k}$:

[12] $$p(n_{k}|x_{k})\approx \frac{{\left(Nx_{k}\right)}^{n_{k}}e^{-Nx_{k}}}{n_{k}!}\;\forall k\in R,$$

[13] $$p(n\prime |x\prime )\approx \frac{\exp\left(-\frac{1}{2}{\left(n\prime -N\prime x\prime \right)}^{T}\Sigma \prime^{-1}(n\prime -N\prime x\prime )\right)}{\sqrt{{\left(2\pi \right)}^{K\prime }\text{det}(\Sigma \prime )}},$$

where the vectors n′ and x′ only take elements k∉R, whose length is K′=K−|R|, and the covariance matrix $\Sigma \prime_{ij}=N\prime x\prime_{i}(\delta_{ij}-x\prime_{j})$. We can assume independent Poisson distributions for each of the rare alleles precisely because they are rare and the effects of drift are approximately independent of each other, and the constraint of constant population size is imposed on the nonrare alleles through the modified population size N′ and the correlation structure in the covariance matrix Σ′.

## Supplementary Material

Supplementary File

## Data Availability

Simulation code data have been deposited in GitHub (https://github.com/BhavKhatri/MutliplexDriveResistanceSims) (40).
