# Supplementary material for: A theory of resistance to multiplexed gene drive demonstrates the significant role of weakly deleterious natural genetic variation
Source: Proc Natl Acad Sci U S A. 2022 Aug 1;119(32):e2200567119. doi: 10.1073/pnas.2200567119 (PMC9371675; doi:10.1073/pnas.2200567119)
Supplement: Supplementary File [file pnas.2200567119.sapp.pdf]

Supplementary Information: A theory of resistance to multiplexed gene drive demonstrates the significant role of weakly deleterious natural genetic variation

Bhavin S. Khatri[a,b,1] **and Austin Burt**[a]

[a]Department of Life Sciences, Imperial College London,Silwood Park, Ascot, SL5 7PY, United Kingdom; [b]The Francis Crick Institute, 1 Midland Road, London, NW1 1AT, United Kingdom

Methods

Single guide RNA. Simulations with a single guide RNA (gRNA) or target site require specification of a 4×4 fitness matrix for both males $\boldsymbol{W}_\mathrm{m}$ and females $\boldsymbol{W}_\mathrm{f}$, whose elements w_{ij} are the fitness of the genotype i/j, where for example genotype $1/3 \equiv \mathsf{W/N}$. In the absence of mutation and drive the allele frequency vector in next generation of females $\boldsymbol{x}_{t+1}$ and males $\boldsymbol{y}_{t+1}$ can be expressed succinctly in matrix notion in terms of the frequency vectors in the current generation $\boldsymbol{x}_t$ and $\boldsymbol{y}_t$ as follows

$$\boldsymbol{x}_{t+1} = \frac{(\boldsymbol{W}_\mathrm{f}\boldsymbol{y}_t)\odot\boldsymbol{x}_t + (\boldsymbol{W}_\mathrm{f}\boldsymbol{x}_t)\odot\boldsymbol{y}_t}{2\bar{w}_\mathrm{f}}, \quad \text{[S1]}$$

$$\boldsymbol{y}_{t+1} = \frac{(\boldsymbol{W}_\mathrm{m}\boldsymbol{x}_t)\odot\boldsymbol{y}_t + (\boldsymbol{W}_\mathrm{m}\boldsymbol{y}_t)\odot\boldsymbol{x}_t}{2\bar{w}_\mathrm{m}}, \quad \text{[S2]}$$

where the mean fitness of males and females is given by the quadratic forms

$$\bar{w}_\mathrm{f} = \boldsymbol{x}_t^T\boldsymbol{W}_\mathrm{f}\boldsymbol{y}_t \quad \text{[S3]}$$

$$\bar{w}_\mathrm{m} = \boldsymbol{y}_t^T\boldsymbol{W}_\mathrm{m}\boldsymbol{x}_t \quad \text{[S4]}$$

and where the $\odot$ operation is the element by element (Hadamard) multiplication operation, i.e. $\boldsymbol{z} = \boldsymbol{x}\odot\boldsymbol{y}$ is equivalent to $z_i = x_i y_i$.

The fitness matrix for males, $\boldsymbol{W}_\mathrm{m}$ is a 4×4 matrix, where each entry, $(W_\mathrm{m})_{ij} = 1$. For females, we assume all functional genotypes (exc N and D) have fitness $w = (1-\sigma)^{n_R}$, where n_R is the number of R alleles in the genotype, so $w_{11} = w(\mathsf{W/W}) = 1$, $w_{12} = w_{21} = w(\mathsf{W/R}) = (1-\sigma)$ and $w_{22} = w(\mathsf{R/R}) = (1-\sigma)^2$. On the other hand for non-functional heterozygotes, we assume a dominance coefficient h with drive to represent somatic leaky expression affecting viability, with a typical value $h \approx 0.3$, and a very recessive dominance coefficient ($h_N = 0.02$) with non-functional resistance alleles; heuristically, this leads to the fitness of genotypes that include functional and non-functional alleles of the form $w = (1-\sigma)^{n_R}(1-h_j s)$, where as before n_R is the number of R alleles in the genotype and if $j = \mathsf{N}$, $h_j = h_N$ and if $j = \mathsf{D}$, $h_j = h$. Genotypes with only non-functional alleles N and D are very deleterious and have fitness $w = 1 - s$, where $s = 1$. Altogether, this gives the following fitness matrix $\boldsymbol{W}_\mathrm{f}$ for females:

$$\boldsymbol{W}_\mathrm{f} = \begin{pmatrix} 1 & 1-\sigma & 1-h_N s & 1-hs \\ 1-\sigma & (1-\sigma)^2 & (1-\sigma)(1-h_N s) & (1-\sigma)(1-h_N s) \\ 1-h_N s & (1-\sigma)(1-h_N s) & 1-s & 1-s \\ 1-hs & (1-\sigma)(1-h_N s) & 1-s & 1-s \end{pmatrix}. \quad \text{[S5]}$$

Including mutations is straightforward using a mutation probability matrix $\boldsymbol{M}$, whose elements M_{ij} represent the probability of generating allele i per generation from allele j, where this stochastic matrix, which acts on probability/frequency *column* vectors must have the property $\sum_i M_{ij} = 1$:

$$\boldsymbol{x}_{t+1} = \boldsymbol{M}\frac{(\boldsymbol{W}_\mathrm{f}\boldsymbol{y}_t)\odot\boldsymbol{x}_t + (\boldsymbol{W}_\mathrm{f}\boldsymbol{x}_t)\odot\boldsymbol{y}_t}{2\bar{w}_\mathrm{f}}, \quad \text{[S6]}$$

$$\boldsymbol{y}_{t+1} = \boldsymbol{M}\frac{(\boldsymbol{W}_\mathrm{m}\boldsymbol{x}_t)\odot\boldsymbol{y}_t + (\boldsymbol{W}_\mathrm{m}\boldsymbol{y}_t)\odot\boldsymbol{x}_t}{2\bar{w}_\mathrm{m}}, \quad \text{[S7]}$$

We make the assumption that there are only two mutational paths $\mathsf{W} \rightarrow \mathsf{R}$ and $\mathsf{W} \rightarrow \mathsf{N}$, which happen with rate $\xi\mu$ and $(1-\xi)\mu$, respectively and there are no backmutations to W (Fig.S1). This gives a mutation matrix:

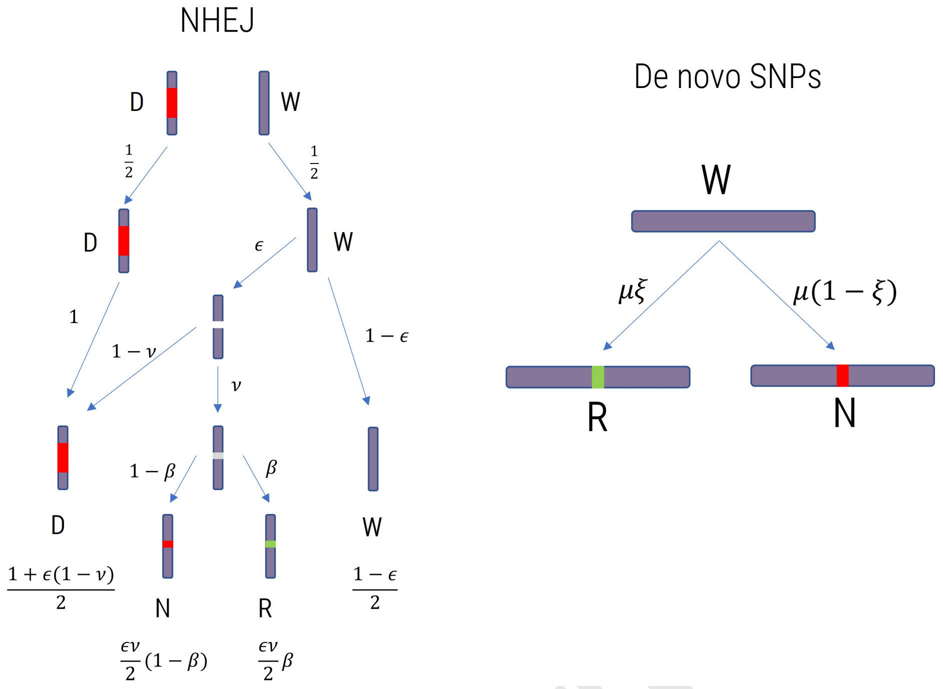

Fig. S1. Diagram showing gametogenesis for $m = 1$ gRNAs with NHEJ for a W/D individual (left) and how de novo SNPs arise by mutation (right). On the left, wild type is the background colour, a white marker indicates the cleavage state, a long red bar represents drive inserted at the target site, a grey marker is an NHEJ event, which are functional (green) or non-functional (red).

DRAFT

$$\boldsymbol{M} = \begin{pmatrix} 1-\mu & 0 & 0 & 0 \\ \xi\mu & 1 & 0 & 0 \\ (1-\xi)\mu & 0 & 1 & 0 \\ 0 & 0 & 0 & 1 \end{pmatrix} \qquad \text{[S8]}$$

Finally, we add the effect of drive by following (S1), where we assume drive acts during meiosis after viability selection. Drive is only produced by heterozygotes W/D, whose frequency after selection and mutation is $(1-\mu)w^{\mathrm{s}}_{14}/\bar{w}_{\mathrm{s}}(x_1y_4 + x_4y_1)$ for females and males, where $\mathrm{s} = \{\mathrm{f}, \mathrm{m}\}$, respectively, which gives the following allele frequency update equations

$$\begin{aligned} \boldsymbol{x}_{t+1} = \boldsymbol{M}\frac{(\boldsymbol{W}_{\mathrm{f}}\boldsymbol{y}_t)\odot\boldsymbol{x}_t + (\boldsymbol{W}_{\mathrm{f}}\boldsymbol{x}_t)\odot\boldsymbol{y}_t}{2\bar{w}_{\mathrm{f}}} \\ + (1-\mu)\frac{w^{\mathrm{f}}_{14}}{2\bar{w}_{\mathrm{f}}}\boldsymbol{\kappa}^{\mathrm{W}}H_t, \end{aligned} \qquad \text{[S9]}$$

$$\begin{aligned} \boldsymbol{y}_{t+1} = \boldsymbol{M}\frac{(\boldsymbol{W}_{\mathrm{m}}\boldsymbol{x}_t)\odot\boldsymbol{y}_t + (\boldsymbol{W}_{\mathrm{m}}\boldsymbol{y}_t)\odot\boldsymbol{x}_t}{2\bar{w}_{\mathrm{m}}} \\ + (1-\mu)\frac{w^{\mathrm{m}}_{14}}{2\bar{w}_{\mathrm{m}}}\boldsymbol{\kappa}^{\mathrm{W}}H_t. \end{aligned} \qquad \text{[S10]}$$

where $H_t = (x_1)_t(y_4)_t + (x_4)_t(y_1)_t$ is the drive-heterozygote frequency (before viability selection acts) and the vector $\boldsymbol{\kappa}^{\mathrm{W}}$ is a stoichiometry-like vector describing the change in fractions of each alleles produced as gametes from the W/D heterozygote relative to no drive:

$$\boldsymbol{\kappa}^{\mathrm{W}} = (-\epsilon, \epsilon\nu\beta, \epsilon\nu(1-\beta), \epsilon(1-\nu))^T. \qquad \text{[S11]}$$

Eqns S9 & S10 are then treated as the mean or expected frequency in a Wright-Fisher multinomial sampling process. To allow for fluctuations in relative numbers of males and females, we perform Wright-Fisher sampling with total population size constraint N_{t+1} on the population vector of female and male allele frequencies $\boldsymbol{z}_{t+1} = (\boldsymbol{x}^T_{t+1}F_{t+1}/N_{t+1}, \boldsymbol{y}^T_{t+1}M_{t+1}/N_{t+1})^T$,

index	haplotype
1	WW
2	WR
3	RR
4	WN
5	RN
6	NN
7	DD

Table 1. Mapping between frequency vector index and haplotypes for 2-fold multiplex

index	haplotype
1	WWW
2	WWR
3	WRR
4	RRR
5	WWN
6	WRN
7	RRN
8	WNN
9	RNN
10	NNN
11	DDD

Table 2. Mapping between frequency vector index and haplotypes for 3-fold multiplex

DRAFT

where now $\boldsymbol{z}_{t+1}$ is a 8 column vector, and for example z_3 is the frequency of female R alleles, and z_5 is the frequency of male W alleles, where F_{t+1}, M_{t+1} and N_{t+1} are the total number of females, males and their sum, which are calculated using a Beverton-Holt scheme described next.

To couple the population genetics calculated here to the population dynamics, we use the Beverton-Holt model of density-dependent growth for male and female populations separately, assuming only females give birth:

$$F_{t+1} = \frac{R_m \bar{w}_{\mathrm{f}} F_t}{1 + N_t/\alpha}, \qquad \text{[S12]}$$

$$M_{t+1} = \frac{R_m \bar{w}_{\mathrm{m}} F_t}{1 + N_t/\alpha}, \qquad \text{[S13]}$$

here R_m is the absolute mean number of offspring from females, and α is a parameter controlling density dependent growth, which we typically tune to give a carrying capacity $K = (R_m(\bar{w}_{\mathrm{f}} + \bar{w}_{\mathrm{m}}) - 1)\alpha = (R_m - 1)\alpha = N_0$, the initial population size we set for the population in the absence of drive. In these simulations we take a typical value of $R_m = 6$.

Multiplexed drive. In general, notational complexity increases considerably when we consider 2-fold and higher orders of multiplexed drive. We will only consider 2-fold and 3-fold multiplex in this paper, but we will set out a general framework for any order of multiplex m.

Firstly, how many different haplotypes n_H are there for each degree m of multiplex? For $n = 3$ alleles at each site (excluding drive), for 2-fold multiplex, as we calculated above there are $n(n+1)/2 = 6$ haplotypes, which is the number of unordered pairs, corresponding to the number of upper diagonal elements for $n \times n$ matrix; including drive, we then need to track a total of $n_H = 7$ haplotype frequencies. For 3-fold multiplex, the number of haplotypes to be tracked is the number of unordered triplets, which is not so trivially calculated, but amounts to calculating the analogue of the number of "upper diagonal" elements of a $n \times n \times n$ tensor. The answer is that there are $n^{(m)}/m! = 3^{(3)}/3! = 10$ haplotypes, excluding drive, for $m = 3$ fold multiplex, where $n^{(m)} = n(n+1)(n+2)...(n+m-1)$ is the rising factorial — note this expression holds true for any m integer. So including drive, for 3-fold multiplex, there are a total of $n_H = 11$ haplotypes frequencies to be tracked.

We also need to decide on the ordering of the haplotypes in the frequency vectors $\boldsymbol{x}$ and $\boldsymbol{y}$. The convention we use is column ordering and its generalisation for higher dimensional tensors, which gives the mapping of indices to haplotypes as shown in Table 1 and Table 2

The fitness matrices for 2-fold multiplex are 7×7, while for 3-fold they are 11×11. For males their fitness matrices are filled with all ones. We do not explicitly write out the fitness matrix of female genotypes for multiplex drive, but its construction follows the same rules as for 1-fold drive described above, where there are 3 categories of genotypes, where now any haplotype with an N at any site becomes non-functional: 1) only functional haplotypes on both chromosomes, where fitness $w = (1-\sigma)^{n_R}$, where n_R is number of R alleles across both chromosomes (e.g. genotype WR/RR has $n_R = 3$, and $w_{23} = w_{32} = (1-\sigma)^3$); 2) functional/non-functional genotypes have fitness of the form $w = (1-\sigma)^{n_R}(1-h_j s)$ (e.g. genotype WRN/RRR has $n_R = 3$ and $h_j = h_N$, so $w_{46} = w_{64} = (1-\sigma)^3(1-h_N s)$, or genotype WWR/DDD has $n_R = 1$ and $h_j = h$, so $w_{2,11} = w_{11,2} = (1-\sigma)(1-hs)$); 3) non-functional genotypes all have fitness $w = 1-s$ (e.g. $w(\mathsf{WWN/DDD}) = w_{5,11} = w_{11,5} = 1-s$).

genotype	heterozygote frequency
WW/DD	$H_1 = x_1 y_7 + x_7 y_1$
WR/DD	$H_2 = x_2 y_7 + x_7 y_2$
WN/DD	$H_4 = x_4 y_7 + x_7 y_4$

Table 3. Drive heterozygotes for 2-fold multiplex and their frequencies. Time dependence is implicit.

genotype	heterozygote frequency
WWW/DDD	$H_1 = x_1 y_{11} + x_{11} y_1$
WWR/DDD	$H_2 = x_2 y_{11} + x_{11} y_2$
WRR/DDD	$H_3 = x_3 y_{11} + x_{11} y_3$
WWN/DDD	$H_5 = x_5 y_{11} + x_{11} y_5$
WRN/DDD	$H_6 = x_6 y_{11} + x_{11} y_6$
WNN/DDD	$H_8 = x_8 y_{11} + x_{11} y_8$

Table 4. Drive heterozygotes for 3-fold multiplex and their frequencies. Time dependence is implicit.

The mutation matrix, for mutiplexed drive of degree m, $\boldsymbol{M}^{(m)}$, is also a $n_H \times n_H$ matrix, the jth column representing the rate of mutations from the jth haplotype to each of the other haplotypes. We assume that the only mutations possible, at each target site, are W $\to$ R and W $\to$ N, with no backmutations. For this reason the only non-zero columns are haplotypes j that have at least a single W allele; of these each W alleles will mutate according the first column of the 1-fold mutation matrix, $\boldsymbol{M}$ (Eqn.S8), whilst the alleles that are not wild type are unaffected by mutation and so are given by the second and third columns of $\boldsymbol{M}$. For mutation probability from the jth haplotype to the ith, if we let n_{W} be the number of W alleles in the jth haplotype, and n'_{W}, n'_{R} and n'_{N} be the number of W, R, N alleles in the subset of the ith haplotype that only has W alleles, then for $m = 2$ the probability of mutation is

$$M^{(2)}_{ij} \equiv M^{(2)}_{k'_1 k'_2, k_1 k_2} = \frac{n_{\mathsf{W}}!}{n'_{\mathsf{W}}! n'_{\mathsf{R}}! n'_{\mathsf{N}}!} M_{k'_1 k_1} M_{k'_2 k_2} \qquad \text{[S14]}$$

where haplotype $k_1 k_2$ maps to j and haplotype $k'_1 k'_2$ maps to i, using Table 1 and k_1, k_2, k'_1, k'_2 can take values $\{1, 2, 3\}$ corresponding to alleles $\{\mathsf{W}, \mathsf{R}, \mathsf{N}\}$, respectively. The multinomial coefficient accounts for the redundancy in the number of ways haplotype $k'_1 k'_2$ can be obtained by mutation of $k_1 k_2$. Explicitly, evaluating this for $m = 2$ we arrive at the following mutation matrix

$$\boldsymbol{M}^{(2)} = \begin{pmatrix} (1-\mu)^2 & 0 & 0 & 0 & 0 & 0 & 0 \\ 2\xi\mu(1-\mu) & 1-\mu & 0 & 0 & 0 & 0 & 0 \\ \xi^2\mu^2 & \xi\mu & 1 & 0 & 0 & 0 & 0 \\ 2(1-\xi)\mu(1-\mu) & 0 & 0 & 1-\mu & 0 & 0 & 0 \\ 2\xi(1-\xi)\mu^2 & (1-\xi)\mu & 0 & \xi\mu & 1 & 0 & 0 \\ (1-\xi)^2\mu^2 & 0 & 0 & (1-\xi)\mu & 0 & 1 & 0 \\ 0 & 0 & 0 & 0 & 0 & 0 & 1 \end{pmatrix} \qquad \text{[S15]}$$

For $m = 3$, equivalent considerations give the following mutation probability from the jth to i^{th} haplotype

$$M^{(3)}_{ij} \equiv M^{(3)}_{k'_1 k'_2 k'_3, k_1 k_2 k_3} = \frac{n_{\mathsf{W}}!}{n'_{\mathsf{W}}! n'_{\mathsf{R}}! n'_{\mathsf{N}}!} M_{k'_1 k_1} M_{k'_2 k_2} M_{k'_3 k_3}, \qquad \text{[S16]}$$

where haplotype $k_1 k_2 k_3$ maps to j and haplotype $k'_1 k'_2 k'_3$ maps to i, using Table 2 and $k_1, k_2, k_3, k'_1, k'_2, k'_3$ can take values $\{1, 2, 3\}$ corresponding to alleles $\{\mathsf{W}, \mathsf{R}, \mathsf{N}\}$, respectively.

For multiplex drive, we make the assumption that drive acts independently at each target site, as shown in Fig. S2 for $m = 2$ gRNAs, and the genotype WW/DD, where we assume the same values of ϵ, ν, and β at each target site; this assumes that cleavage does not act simultaneously across target sites and that there are no dosage effects of Cas9 with increasing number of gRNAs/target sites (S2, S3). The fraction of gametes is then effectively calculated by a multiplicative table of the fractions of W, R, N, D produced at each target site; since only a single successful cleavage and repair is needed (signified by a single D), we need to account for all fractions shown shaded in red in the table, to calculate the total fraction of DD gametes. This is formalised mathematically below and extended to any number of m of gNRAs.

To consider how multiplexed drive affects haplotype frequencies, we need to consider now that there is more than just a single genotype that is affected by drive. All genotypes with drive on one chromosome and at least a single W allele on the other can be converted to homozygous drive; for $m = 2$ there are three which are enumerated in Table 3 and for $m = 3$ there are six enumerated in Table 4.

Each of these heterozygotes produces different fractions of the n_H possible haplotypes, depending on the number of W alleles in each haplotype. We create an analogous matrix to $\boldsymbol{M}$ (Eqn. S8), whose j^{th} column gives the fraction of gametes produced by the jth allele assuming it is in genotype with D:

DRAFT

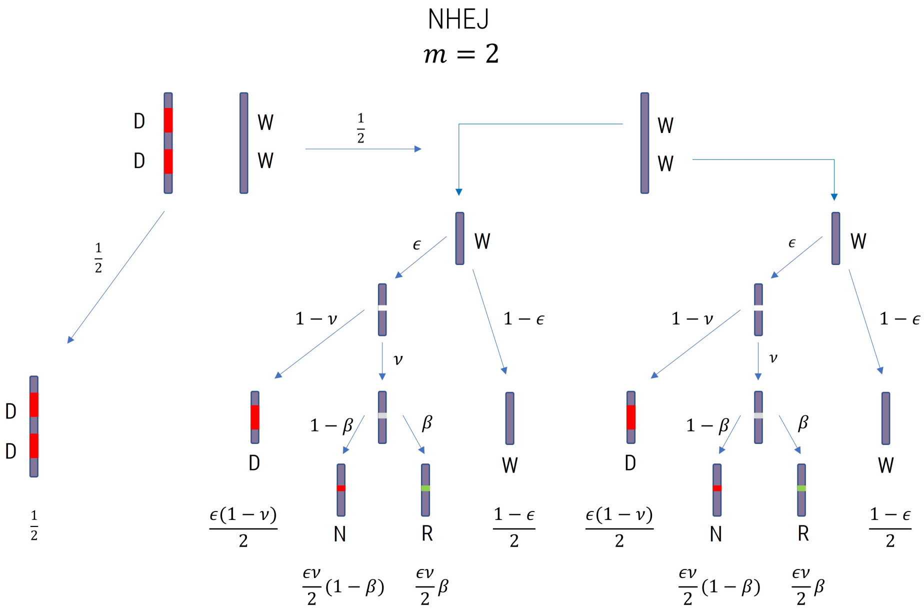

	W	R	N	D
W	$\frac{1}{4}(1-\epsilon)^2$	$\frac{1}{4}(1-\epsilon)\epsilon\nu\beta$	$\frac{1}{4}(1-\epsilon)\epsilon\nu(1-\beta)$	$\frac{1}{4}(1-\epsilon)\epsilon(1-\nu)$
R	$\frac{1}{4}(1-\epsilon)\epsilon\nu\beta$	$\frac{1}{4}(\epsilon\nu\beta)^2$	$\frac{1}{4}(\epsilon\nu)^2\beta(1-\beta)$	$\frac{1}{4}\epsilon^2\nu(1-\nu)\beta$
N	$\frac{1}{4}(1-\epsilon)\epsilon\nu(1-\beta)$	$\frac{1}{4}(\epsilon\nu)^2\beta(1-\beta)$	$\frac{1}{4}(\epsilon\nu(1-\beta))^2$	$\frac{1}{4}\epsilon^2\nu(1-\nu)(1-\beta)$
D	$\frac{1}{4}(1-\epsilon)\epsilon(1-\nu)$	$\frac{1}{4}\epsilon^2\nu(1-\nu)\beta$	$\frac{1}{4}\epsilon^2\nu(1-\nu)(1-\beta)$	$\frac{1}{4}\epsilon^2(1-\nu)^2$

Total fraction of DD gametes $\frac{1}{2}$ + all terms shaded in red

Fig. S2. Diagram showing gametogenesis for $m = 2$ gRNAs with NHEJ for a W/D individual, where the model assumes the action of drive is independent on each target site. wild type is the background colour, a white marker indicates the cleavage state, a long red bar represents drive inserted at the target site, a grey marker is an NHEJ event, which are functional (green) or non-functional (red).

$$\boldsymbol{\kappa} = \begin{pmatrix} 1-\epsilon & 0 & 0 & 0 \\ \epsilon\nu\beta & 1 & 0 & 0 \\ \epsilon\nu(1-\beta) & 0 & 1 & 0 \\ \epsilon(1-\nu) & 0 & 0 & 1 \end{pmatrix}. \quad \text{[S17]}$$

Note that the first column of $\boldsymbol{\kappa}$ is $\boldsymbol{\kappa}^{\mathsf{W}}$, but with $\boldsymbol{\kappa}_1^{\mathsf{W}} \to \boldsymbol{\kappa}_1^{\mathsf{W}} + 1$.

With this matrix the fraction of gametes that have the ith haplotype from drive heterozygote j can be calculated for any value of m. For haplotypes $i < n_H$ (i.e. not including drive) this is given by

$$\boldsymbol{\kappa}_{ij}^{(m)} = \frac{n_{\mathsf{W}}!}{n'_{\mathsf{W}}!n'_{\mathsf{R}}!n'_{\mathsf{N}}!} \prod_{\ell}^{m} \kappa_{k'_\ell k_\ell} - \delta_{ij} \qquad (i < n_H), \quad \text{[S18]}$$

where n_{W} is the number of W alleles in the drive heterozygote j, and $n'_{\mathsf{W}}, n'_{\mathsf{R}}, n'_{\mathsf{N}}$ are the number of W, R, N alleles in haplotype i, and as before the haplotypes k_1k_2 and $k'_1k'_2$ map to indices using Table 1. For $i = n_H$, the drive haplotype (i.e. DD or DDD), we need to sum over all haplotypes i that produce at least a single D allele (which is effectively converted to, for example, DD or DDD); if these set of haplotypes is denoted by $\mathcal{D}_j$ then

$$\boldsymbol{\kappa}_{n_H,j}^{(m)} = \sum_{i\in\mathcal{D}_j} \frac{n_{\mathsf{W}}!}{n'_{\mathsf{W}}!n'_{\mathsf{R}}!n'_{\mathsf{N}}!n_{\mathsf{D}}!} \prod_{\ell}^{m} \kappa_{k'_\ell k_\ell} - \delta_{n_H,j}, \quad \text{[S19]}$$

where on the RHS the relation between $i \in \mathcal{D}_j$ and k'_ℓ, k'_ℓ, $n'_{\mathsf{W}}, n'_{\mathsf{R}}, n'_{\mathsf{N}}$ and n'_{D} is implicit, and straightforward to enumerate.

Together these equations allow us to write down expressions for the update equations for haplotype frequencies between generations for $m-$fold multiplex

$$\begin{aligned} \boldsymbol{x}_{t+1} = \boldsymbol{M}^{(m)} \frac{(\boldsymbol{W}_{\mathrm{f}}\boldsymbol{y}_t)\odot \boldsymbol{x}_t + (\boldsymbol{W}_{\mathrm{f}}\boldsymbol{x}_t)\odot \boldsymbol{y}_t}{2\bar{w}_{\mathrm{f}}} \\ + \sum_{j\in\mathcal{H}_m} \frac{w^{\mathrm{f}}_{j,n_H}}{2\bar{w}_{\mathrm{f}}} \boldsymbol{\kappa}^{(m)}_{:,j} H_j M^{(m)}_{jj}, \end{aligned} \quad [\mathrm{S20}]$$

$$\begin{aligned} \boldsymbol{y}_{t+1} = \boldsymbol{M}^{(m)} \frac{(\boldsymbol{W}_{\mathrm{m}}\boldsymbol{x}_t)\odot \boldsymbol{y}_t + (\boldsymbol{W}_{\mathrm{m}}\boldsymbol{y}_t)\odot \boldsymbol{x}_t}{2\bar{w}_{\mathrm{m}}} \\ + \sum_{j\in\mathcal{H}_m} \frac{w^{\mathrm{m}}_{j,n_H}}{2\bar{w}_{\mathrm{m}}} \boldsymbol{\kappa}^{(m)}_{:,j} H_j M^{(m)}_{jj}, \end{aligned} \quad [\mathrm{S21}]$$

where $\mathcal{H}_m$ is the set of indices of haplotypes that have at least a single W allele, which is a function of m the degree of multiplexing (Tables 3&4), H_j are the drive-heterozygote frequencies given in Tables 3&4, where the time-dependence on t is implicit, and where the notation $\boldsymbol{\kappa}^{(m)}_{:,j}$ is the jth column of the matrix $\boldsymbol{\kappa}^{(m)}$.

Typical trajectories of allele/haplotype frequencies. In Fig. S3 we show for $m = 1$, $m = 2$ and $m = 3$ the typical trajectories of the different alleles/haplotypes at population sizes for $N < N^*$ (left hand plots of the figure), where resistance does not arise and $N > N^*$ (right hand plots of the figure), where resistance does arise, due to de novo mutation or NHEJ. Below each is the corresponding time series of population size, showing population elimination (left) and rescue (right). These simulations assume that the fraction of functional NHEJ mutations is $\beta = 10^{-4}$ and that all de novo SNPs are functional ($\xi = 1$). We see in all cases that initially drive replaces the wild type in less than 10 generations, which causes the mean population fitness to decrease and the population size itself to decrease; following this, if $N > N^*$ we see that functional resistance mutants arise and then fix giving rise to population rescue, while for $N < N^*$, resistance mutants do not arise on the timescale at which the population is eliminated. As we increase the degree of multiplexing m, the same story unfolds, except there are many more resistance haplotypes, which all arise at small frequency, and except for the single functional mutant for each value of m (R, RR and RRR, respectively for $m = 1, 2, \&3$ gRNAs), none of these rise to sufficiently high frequency to achieve population rescue as they are deleterious as homozygotes, and as drive heterozygotes, haplotypes containing W can still be converted to D. The resistance haplotypes that have m copies of R do increase to high frequencies giving resistance and population rescue.

DRAFT

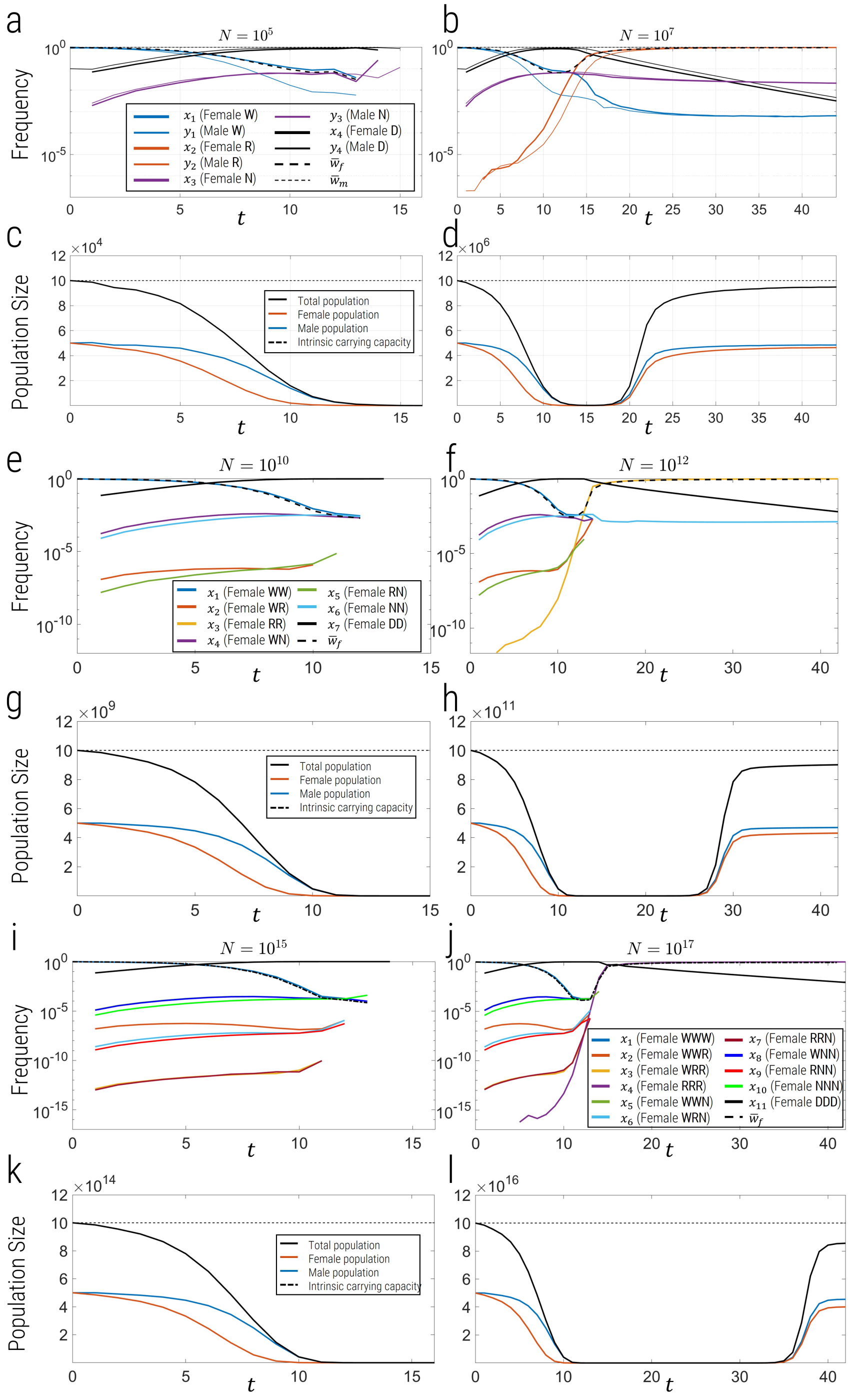

Fig. S3. Plot of frequency of different alleles (a,b, e, f, i & j) and population dynamics (c, d, g, h, k & l) vs generation time for $N < N^*$ (a, c, e, g, i & k) and $N > N^*$ (b, d, h, j & l) for $m = 1$ gRNA (top panel), $m = 2$ gRNAs (middle panel) and $m = 3$ gRNAs (bottom panel), where N^* is the critical population size at which the probability of resistance (rescue) is $1 - e^{-1}$. For $m = \{2, 3\}$, we only plot the female allele frequencies for clarity.

Concurrent vs Sequential accumulation of resistance alleles for NHEJ vs de novo mutation

When there are multiple gRNAs, whether by NHEJ or de novo SNPs, functional resistance mutations can arise sequentially (e.g. WWW $\rightarrow$ WWR $\rightarrow$ WRR $\rightarrow$ RRR) or concurrently through m-fold mutations (e.g. WWW $\rightarrow$ RRR) across the target sites, or some combination of the two (e.g. WWW $\rightarrow$ WWR $\rightarrow$ RRR). Here, using a simple heuristic analysis, we examine based on calculating the probability of fixation when there are a certain number of copies of functional resistance haplotypes, what signature we would expect in the scaling of N^* with respect to the base rate of generation of each type of mutant, which is $\epsilon\beta\nu$

for NHEJ and $\xi\mu$ for de novo SNPs. The approach essentially deterministically calculates the number of mutants generated over a certain timescale and ignores all fitness effects, assuming drift dominates at small frequencies, and only accounts for the fitness through the probability of establishment.

The results below indicate that in general 1) we expect resistance mutations to arrive concurrently for NHEJ vs sequentially for de novo SNPs and 2) for the same individual rate of producing functional mutants at all m sites, the critical population size for de novo generation of mutants is smaller, and it is easier for resistance to arise de novo compared to NHEJ generation of mutants and the disparity increases as the number of gRNAs m increases. This second effect, however, will be largely masked by the generally much higher rate of generating NHEJ mutants compared to de novo SNPs.

Generation of functional resistance non-homologous end joining mutants (NHEJ). For a single gRNA, per generation and per each heterozygous W/D individual, non-homologous end joining mutants arise at a rate proportional to $\epsilon\nu$, and a fraction of β of these we assume to be functional (R), and so the rate of producing functional NHEJ mutants is proportional to $\epsilon\nu\beta$. Hence, if r_t is the frequency of the R allele in the population in generation t, then the difference equation describing the dynamics is given by

$$r(t+1) = r(t) + \epsilon\nu\beta q(t)(1-q(t)), \quad \text{[S22]}$$

where q_t is the frequency of the D allele and to a good approximation $q(t)(1-q(t))$ is the frequency of the W/D heterozygotes, assuming all other alleles apart from the W and D are at much smaller frequencies initially. Mutants generated earlier will generally have contribute most to the ultimate probability of establishment and population rescue, and so we make the approximation that the frequency of the heterozygotes is roughly $q(t)$ and so

$$r(t+1) \approx r(t) + \epsilon\nu\beta q(t), \quad \text{[S23]}$$

which has solution $r(t) = r(0) + \epsilon\nu\beta Q(t) = \epsilon\nu\beta Q(t)$, since we assume at $t = 0$ when drive is introduced there are no mutants, and where $Q(t) = \sum_{t'=0}^{t-1} q(t')$. If we assume there is some characteristic time over which mutants are generated, related to the time to fixation of drive τ, then the number of copies of the mutant generated in this time is τ is therefore $2Nr(\tau) = 2N\epsilon\nu\beta Q(\tau)$. If each mutant has probability of fixation of $\pi = 2s_b$, where s_b is an effective selection coefficient of the benefit the functional resistance allele has in the presence of drive, then given this number of mutants, we calculate the probability of fixation by 1-probability that none of these mutants establishes:

$$\begin{aligned} p &= 1-(1-\pi)^{Nr(\tau)} \\ &\approx 1-e^{-\pi N r(\tau)} \\ &= 1-e^{-4Ns_b\epsilon\nu\beta Q(\tau)} \\ &= 1-e^{-N/N_n^*} \end{aligned} \quad \text{[S24]}$$

where $N_n^* = (4s_b\epsilon\nu\beta Q(\tau))^{-1}$, which gives us the result that we expect resistance to arise significantly, when $N > N_n$ or the rate of generation of NHEJ mutants is $2N\epsilon\nu\beta \sim (2s_bQ(\tau))^{-1}$, then the probability of resistance is large.

For $m = 2$ gRNAs, a single resistance mutation (WR or RW) is not sufficient to prevent the copying of drive and so only haplotypes with two functional resistance mutations (RR) have a selective advantage in the presence of drive. Assuming the frequency of all mutants is small, and so assuming the fact that single mutants are selected against can be ignored (due to drift), the difference equations describing this scenario are

$$r_1(t+1) = r_1(t) + 2(1-\epsilon)\epsilon\nu\beta q(t)(1-q(t)) \quad \text{[S25]}$$

$$r_2(t+1) = r_2(t) + \epsilon\nu\beta r_1(t)(1-q(t)) + (\epsilon\nu\beta)^2 q(t)(1-q(t)) \quad \text{[S26]}$$

where r_1 is the frequency of mutants with a single functional resistance allele at either of the two target sites and r_2 is the frequency of mutants that have a functional resistance allele at both target sites. If we make the same assumption that initially the frequency of drive will be small, such that $q(1-q) \approx q$, then the solution for the frequency of single-fold functional resistance mutants is $r_1(t) = 2(1-\epsilon)\epsilon\nu\beta Q(t)$, and so we have a difference equation for only r_2:

$$r_2(t+1) = r_2(t) + 2(1-\epsilon)(\epsilon\nu\beta)^2 Q(t) + (\epsilon\nu\beta)^2 q(t). \quad \text{[S27]}$$

The second term represents the generation of two-fold mutants from single-fold heterozygotes with drive (WR/DD or RW/DD), whilst the third term represents generation directly from wild type-drive heterozygotes. We can see that if $1-\epsilon$ is small then the latter direct mechanism of generation will be dominant, as long as the cumulative frequency of drive $Q(t)$ is not significantly larger than the current frequency $q(t)$; as $Q(t)$ is a cumulative frequency it will be of order $1/s_D$, where $s_D = (1+\epsilon)(1-hs/2)-1$ is the effective selection coefficient of drive, which gives the condition $s_D \gg 2(1-\epsilon)$ such that direct generation of double mutants dominates over sequential generation. For typical parameters of suppression drive as used in this paper $\epsilon = 0.95$, $s = 1$ and $h = 0.3$, we get $s_D \approx 0.66$ and $2(1-\epsilon) = 0.1$, and so we would expect direct generation of double mutants to dominate. If this is the case then our difference equation for double functional mutants is

DRAFT

$$r_2(t+1) \approx (\epsilon\nu\beta)^2 q(t). \quad \text{[S28]}$$

and so comparing to Eqn. S22, we have the same difference equation but $\epsilon\nu\beta \to (\epsilon\nu\beta)^2$ and so would expect the probability of resistance to be of the form $p = 1 - e^{-N/N_n^*}$ with $N_n^* = (4s_b(\epsilon\nu\beta)^2 Q(\tau))^{-1}$. A similar analysis can be performed for $m > 2$ gRNAs to give in general $N_n^* = (4s_b(\epsilon\nu\beta)^m Q(\tau))^{-1}$, for m gRNAs. Here, we see that if we let $\gamma_n = s_b Q(\tau)$ then we have a single unknown constant, which is the same for all m, and fitting to the simulation data of p vs N for different values of β (Fig. 1 in main text), we find indeed this is the case and $\gamma \approx 0.2$ for $m = \{1, 2, 3\}$, as shown in Fig. S4.

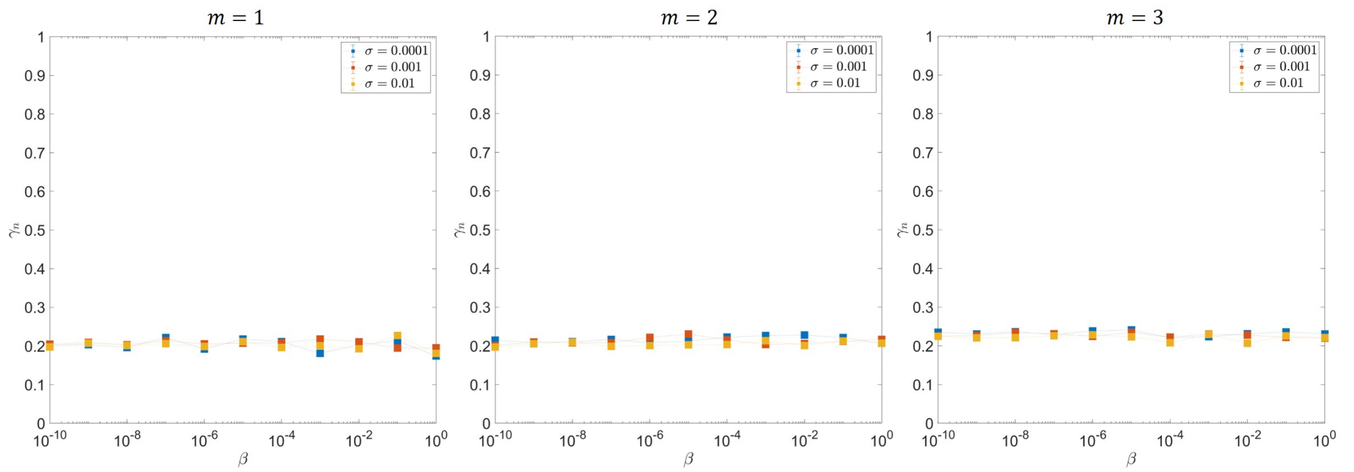

Fig. S4. Values of γ_n fitting parameter from fits to simulation results of probability of resistance p vs N for different values of β, where $p = 1 - e^{-N/N_n^*}$ with $N_n^* = (4\gamma_n(\epsilon\nu\beta)^m)^{-1}$, where each panel are for different numbers of gRNAs m as indicated.

De novo generation of SNP functional resistant mutants. In the case of de novo generation of mutants the difference equation for a single gRNA is

$$r(t+1) = r(t) + \xi\mu(1 - q(t)) \approx r(t) + \xi\mu, \quad \text{[S29]}$$

where we assume that initially $q \ll 1$. The solution is simply $r(t) = \xi\mu t$. Using the same simple heuristic as for NHEJ, this then leads to the probability of fixation of mutants generated up to some time τ, related to the timescale for fixation of drive, to be:

$$\begin{aligned} p &= 1 - (1-\pi)^{Nr(\tau)} \\ &\approx 1 - e^{-\pi N r(\tau)} \\ &= 1 - e^{-4Ns_b\xi\mu\tau} \\ &= 1 - e^{-N/N_d^*} \end{aligned} \quad \text{[S30]}$$

with $N_d^* = (4s_b\xi\mu\tau)^{-1}$.

For $m = 2$ gRNAs, the difference equations for de novo generation of single and double functional resistance mutants is

$$r_1(t+1) = r_1(t) + 2\xi\mu(1 - q(t)) \approx r_1(t) + \xi\mu \quad \text{[S31]}$$

$$r_2(t+1) = r_2(t) + \xi\mu r_1(t) + (\xi\mu)^2(1 - q(t)) \approx r_2(t) + \xi\mu r_1(t) + (\xi\mu)^2 \quad \text{[S32]}$$

where again we assume the frequency of drive is initially small and we ignore any fitness effects for mutants at small frequency in calculating the frequency of mutants. As above the frequency of single mutants will be $r_1(t) \approx 2\xi\mu t$, where now as there are two paths from the wild type to generate a single mutant there is a factor of 2, and so the difference equation for double functional mutants will be

$$r_2(t+1) \approx r_2(t) + 2(\xi\mu)^2 t + (\xi\mu)^2. \quad \text{[S33]}$$

Now here the second term, representing the sequential generation of mutants, will dominate for any sufficiently large number of generations $t > 1$ and so ignoring the third term which represents concurrent generation of mutants, the solution is $r_2(t) = 2(\xi\mu)^2 t(t+1)/2 \sim (\xi\mu)^2 t^2$ for large t. This means on a time scale τ the number of functional double-mutants generated will be $2Nr_2(\tau) \sim 2N \times (\xi\mu)^2 t^2 = 2N(\xi\mu)^2 t^2$. Following the previous arguments, the probability of fixation will

DRAFT

then be $p \approx 1 - e^{-N/N_d^*}$ with $N_d = (4(\xi\mu)^2 s_b \tau^2)^{-1}$. Again the arguments can be extended to $m = 3$ gRNAs and we find $N_d = (4(\xi\mu)^2 s_b \tau^3)^{-1}$, to leading order in τ.

We can fit the simulation data of the de novo probability of resistance vs N as ξ is varied, using this heuristic theory with a single fitting constant γ_m and $N_d^* = (4(\xi\mu)^m \gamma_m)^{-1}$, for each value of m and we find $\gamma_1 = 0.76 \pm 0.004$, $\gamma_2 = 6 \pm 0.04$ and $\gamma_3 = 55 \pm 0.3$, as shown in Fig. S5; using the relation $\gamma_m = s_b \tau^m$, we find these data are consistent with $\tau \approx 8.5$ generations and a beneficial selection coefficient of $s_b \approx 0.09$.

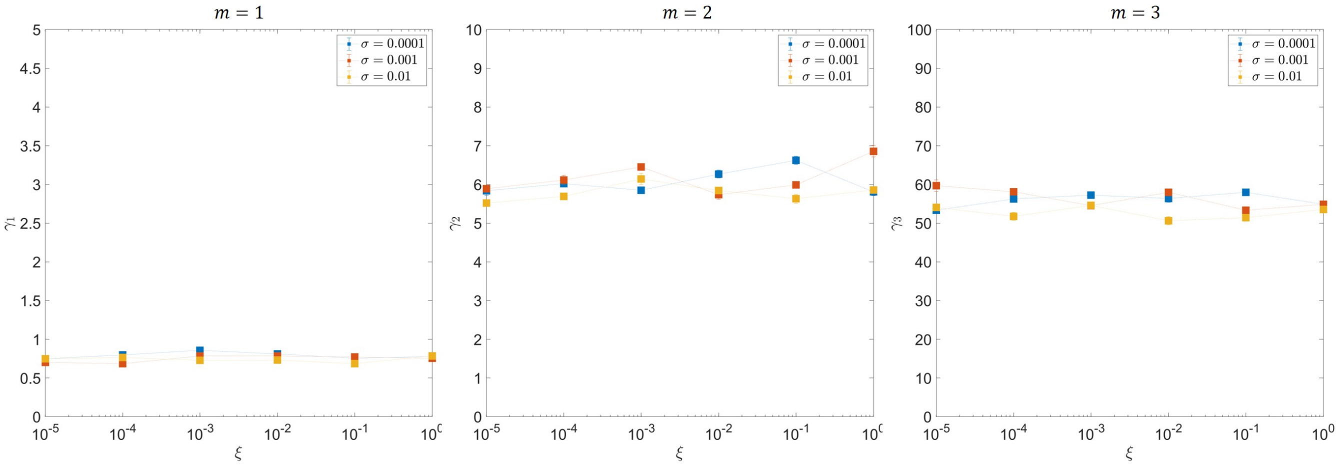

Fig. S5. Values of γ_m fitting parameter from fits to simulation results of probability of resistance p vs N for different values of ξ, where $p = 1 - e^{-N/N_d^*}$ with $N_d^* = (4\gamma_m(\xi\mu)^m)^{-1}$, where each panel are for different numbers of gRNAs m as indicated.

DRAFT

Multiplex resistance from standing variation of SNP mutants. In this section, we show that the significant amplification of standing variation or pre-existing mutants for $m > 1$ gRNAs can be heuristically understood by calculating approximately the average probability of fixation of m-fold functionally resistant, assuming that in the presence of drive it has a beneficial selection coefficient s_b and that before the introduction of drive it is mildly selected against, giving a distribution of the frequency of such mutants in mutation-selection balance.

For $m = 1$ gRNAs the relevant theory has already been calculated in detail by Hermisson & Pennings (S4), which we reproduce here in brevity and slightly simplified form. In mutation-selection balance, the diffusion approximation can be used to calculate the equilibrium distribution of the frequency of such variants. Here a single functional resistance mutant has selection coefficient $-\sigma$ before the introduction of drive and the distribution of frequency r is approximately gamma distributed, as long as $\alpha = 4N\sigma \gg 1$:

$$\rho(r) = \frac{\alpha^\theta}{\Gamma(\theta)} r^{\theta-1} e^{-\alpha r} \tag{S34}$$

which has shape parameter $\theta = 4N\xi\mu$ and rate α. The mean of this distribution is $\langle r \rangle = \theta/\alpha = \xi\mu/\sigma$, which is the classic mutation-selection balance frequency. Now the probability of fixation given an initial frequency r_0 and beneficial selection coefficient s_b is

$$\pi(r_0) = \frac{1 - e^{-\alpha_b r_0}}{1 - e^{-\alpha_b}} \approx 1 - e^{-\alpha_b r_0}, \tag{S35}$$

where the last approximation assumes $\alpha_b = 4Ns_b \gg 1$. The average probability of fixation assuming a distribution $\rho(r_0)$ is then simply calculated by averaging over the initial frequency r_0, which gives

$$\langle \pi \rangle = 1 - \frac{Z(\alpha_b + \alpha, \theta)}{Z(\alpha, \theta)}, \tag{S36}$$

where Z is the normalisation constant of the gamma distribution with population scaled fitness f: $Z(f, \theta) = \int_0^\infty r^{\theta-1} e^{-fr} \mathrm{d}r$, where extending the integral to ∞ will have small penalty in the strong selection limit, i.e. as long as $f \gg 1$. Evaluating this and re-arranging we find the result of Hermisson & Pennings in the strong selection limit:

$$\langle \pi \rangle = 1 - e^{-4N\xi\mu \ln\left(1 + \frac{s_b}{\sigma}\right)} \tag{S37}$$

And so the probability of resistance $p = 1 - e^{-N/N_s^*}$, where $N_s^* = \left(4\xi\mu \ln\left(1 + \frac{s_b}{\sigma}\right)\right)^{-1}$, which is of the same form as for NHEJ and de novo SNP mutations. There is only a single unknown parameter s_b, and so fitting this form to the simulation data

for the p vs N, for varying ξ we find $s_b \approx 0.5$ as shown in Fig. S6 ($m = 1$ panel), which is larger than found from de novo simulations, and likely reflects some mechanistic aspect not reflected in these simple heuristic considerations.

For $m = 2$, we could try to calculate exactly the joint steady-state distribution of the frequency of all mutants, under the sequential mutation scheme $\mathsf{WW} \rightarrow \{\mathsf{WR}, \mathsf{RW}\} \rightarrow \mathsf{RR}$, but this is in general an unsolved problem in population genetics, since there is a net flux or current of probability through the states of the system (detailed balance is not obeyed) and so calculating this joint distribution is not straightforward. Here, instead we appeal to a simple heuristic, which reproduces the scaling with σ seen in the simulation data; we assume the distribution of the double-mutant frequency r_2 follows the same gamma distribution, but that mutation to this allele is solely from single mutants whose frequency is assumed fixed at its equilibrium frequency $\langle r_1 \rangle = 2\xi\mu/\sigma$, where the factor 2 arises from the multiplicity 2 of paths to single mutants from the wild type; this is reasonable given that sequential mutation rather than concurrent is dominant, as demonstrated for de novo mutation. Given this and that the fitness cost of double mutants, before drive is introduced, is -2σ, the distribution of the double functional resistance mutants is

$$\rho_2(r_2) = \frac{{\alpha_2}^{\theta_2}}{\Gamma(\theta_2)} r^{\theta_2 - 1} e^{-\alpha_2 r}, \qquad \text{[S38]}$$

where $\alpha_2 = 8N\sigma$, and $\theta_2 = 4N\xi\mu\langle r_1 \rangle = 8N(\xi\mu)^2/\sigma$. The average frequency of double functional mutants is $\theta_2/\alpha_2 = (\xi\mu)^2/\sigma^2$, which is simple to show is what we would expect solving the deterministic equations for the frequency of double-mutants (see section below). Using the form of Eqn. S36, we find

$$\langle \pi \rangle = 1 - e^{-\frac{8N(\xi\mu)^2}{\sigma} \ln\left(1 + \frac{s_b}{2\sigma}\right)}, \qquad \text{[S39]}$$

which is of the form $1 - e^{-N/N_s^*}$, with $N_s^* = \left(\frac{8(\xi\mu)^2}{\sigma} \ln\left(1 + \frac{s_b}{2\sigma}\right)\right)^{-1}$, which we see means $N_s^* \sim \sigma$ for $m = 2$, instead of the much weaker logarithmic dependence for $m = 1$. A similar analysis can be done for $m = 3$, which gives $N_s^* \sim \left(\frac{(\xi\mu)^3}{\sigma^2} \ln\left(1 + \frac{s_b}{3\sigma_b}\right)\right)^{-1} \sim \sigma^2$, which is consistent with the power law scaling we see in the simulations (Fig. 3b). In practice, we find that the following form $N_s^* = (4\xi\mu m \left(\frac{\gamma_s \xi\mu}{\sigma}\right)^{m-1} \ln\left(1 + \frac{s_b}{m\sigma_b}\right))^{-1}$ fits the simulation data of p vs N for different values of ξ and m with a single fitting parameter $\gamma_s \approx 2$ (for $m = 2$ and $m = 3$) as shown in Fig. S6.

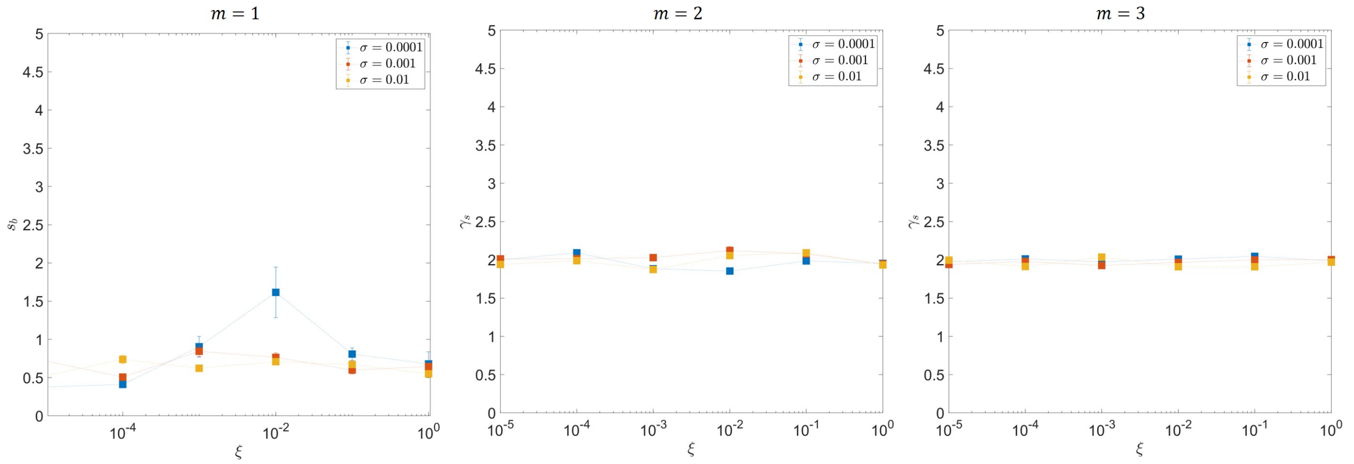

Fig. S6. Values of s_b ($m = 1$) and γ_s ($m = 2$ and $m = 3$) fitting parameter from fits to simulation results of probability of resistance p vs N for different values of ξ with standing variation and de novo SNPs and NHEJ turned off, where $p = 1 - e^{-N/N_s^*}$ with $N_s^* = (4\xi\mu m \left(\frac{\gamma_s \xi\mu}{\sigma}\right)^{m-1} \ln\left(1 + \frac{s_b}{m\sigma_b}\right))^{-1}$, where each panel are for different numbers of gRNAs m as indicated.

Note that there is an implicit assumption in all these calculations, that establishment must arise before the population is removed, which will be true if $\sim 1/s_b$ is much smaller than the timescale over which the population is removed.

Equilibrium frequency of multi-site deterministic mutation selection balance

If before the introduction of drive we assume the wild type is almost fixed and the k-fold resistance haplotype has frequency r_k, that $\sigma \gg \mu$ and $x_{k-1} \gg x_k$ then the ODE for the kth resistance haplotype is

$$\frac{\mathrm{d}r_k}{\mathrm{d}t} = -\sigma x_k(1 - x_k) + \mu(x_{k-1} - x_k) \approx -\sigma x_k + \mu x_{k-1}, \qquad \text{[S40]}$$

where we have ignored combinatorial factors in the rate of mutation between haplotypes with k resistance alleles and the extra fitness cost as k increases. We find the fixed point for this set of couple equations recursively, starting with $r_2^* = \mu/\sigma$ (given the frequency of the wild type is close to 1), to give

$$\frac{\mathrm{d}r_k}{\mathrm{d}t} \approx -\sigma x_k + \frac{\mu^k}{\sigma^{k-1}}, \qquad \text{[S41]}$$

and the equilibrium frequency for r_k^*:

$$r_k^* = \left(\frac{\mu}{\sigma}\right)^k \qquad \text{[S42]}$$

In the stochastic analysis of the previous section this becomes the mean frequency of the k-fold resistance haplotype: $\langle r_k \rangle = r_k^*$.

DRAFT

Time to Resistance

In this section we plot the mean time to resistance over $M = 500$ replicates. We define the time to resistance to be when the sum of the frequencies of resistance haplotypes exceed or equal 0.95. Figs. S7 are simulations for NHEJ only, Figs. S8 are simulations for de novo mutations only, and Figs. S9 are simulations for preexisting and de novo mutations only.

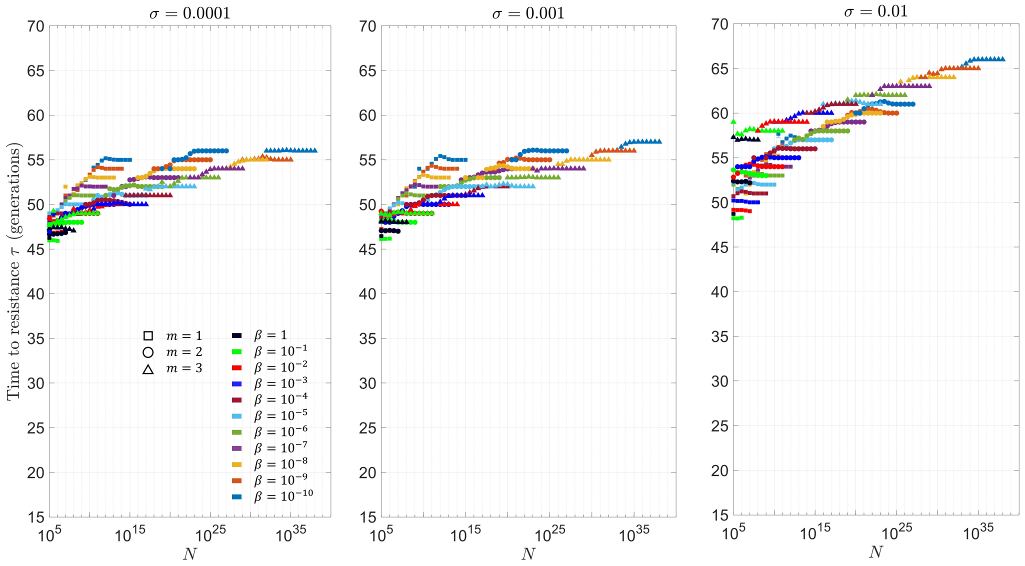

Fig. S7. NHEJ only. Mean time to resistance over $M = 500$ replicate simulations for $m = \{1, 2, 3\}$ gRNA. Standard parameters of simulations.

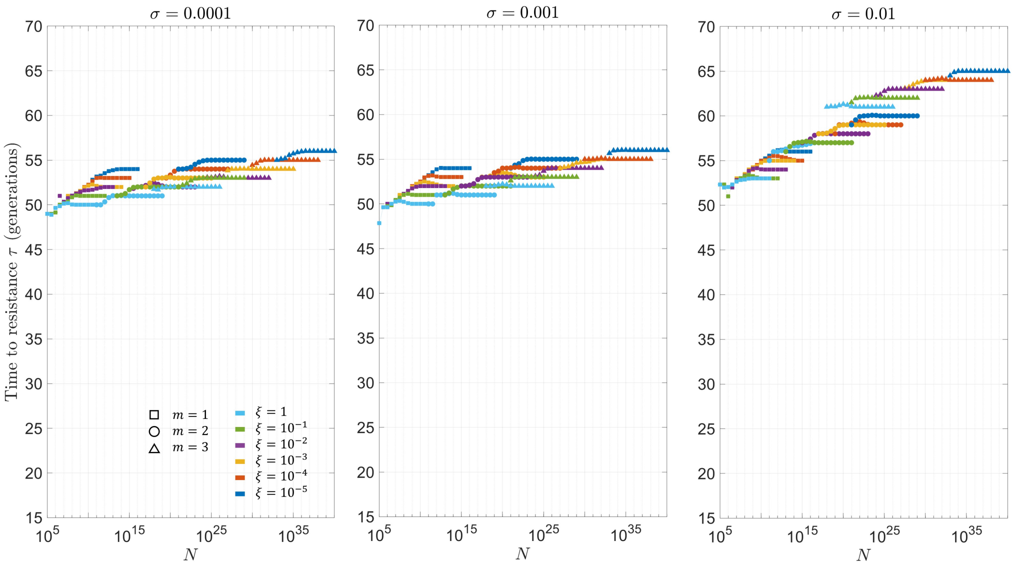

Fig. S8. De novo single nucleotide mutations only. Mean time to resistance over $M = 500$ replicate simulations for $m = \{1, 2, 3\}$ gRNAs. Standard parameters of simulations.

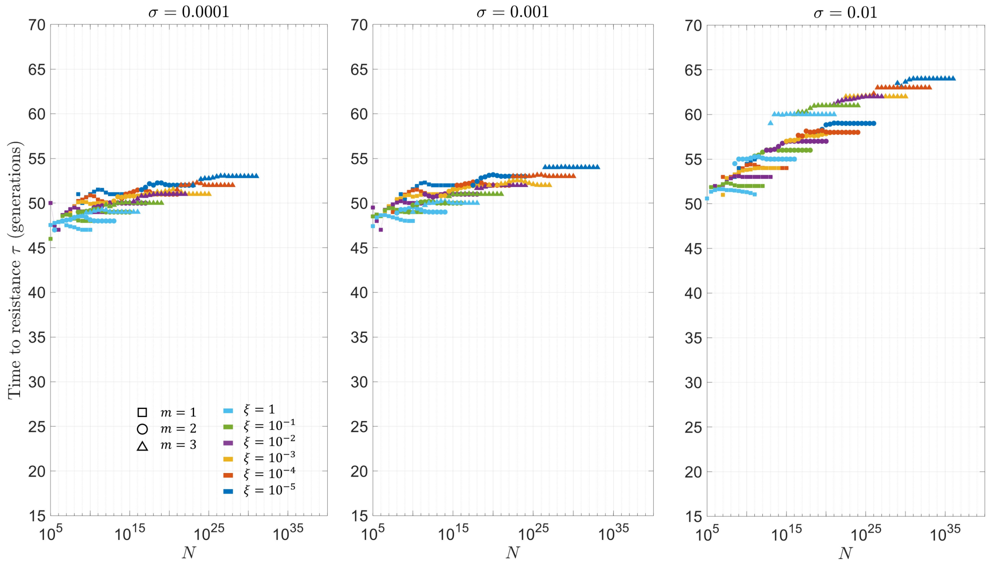

Fig. S9. Single nucleotide mutations only (pre-existing + de novo). Mean time to resistance over $M = 500$ replicate simulations for $m = \{1, 2, 3\}$ gRNAs. Standard parameters of simulations.

References

S1. A Deredec, A Burt, HCJ Godfray, The Population Genetics of Using Homing Endonuclease Genes in Vector and Pest Management. *Genetics* **179**, 2013–2026 (2008).
S2. TAA Prowse, et al., Dodging silver bullets: good CRISPR gene-drive design is critical for eradicating exotic vertebrates. *Proc. Royal Soc. B: Biol. Sci.* **284**, 20170799 (2017).
S3. SE Champer, et al., Computational and experimental performance of CRISPR homing gene drive strategies with multiplexed gRNAs. *Sci. Adv.* **6**, eaaz0525 (2020).
S4. J Hermisson, PS Pennings, Soft sweeps: molecular population genetics of adaptation from standing genetic variation. *Genetics* **169**, 2335–2352 (2005).

DRAFT
